# Executive Function Correlates of Women Victims of Intimate Partner Violence: A Systematic Review and Meta-Analysis

**DOI:** 10.1177/15248380251381820

**Published:** 2025-11-12

**Authors:** Sara Ferreira-Nascimento, Miguel Rodrigues Barbosa, Magda Sofia Roberto, Sandra Fernandes, Rute Pires, Luís Querido

**Affiliations:** 1 Faculty of Psychology, University of Lisbon, Alameda da Universidade, Lisbon, Portugal; 2 CICPSI – Research Center for Psychological Science, Faculty of Psychology, University of Lisbon, Alameda da Universidade, Lisbon, Portugal; 3 Egas Moniz Center for Interdisciplinary Research (CiiEM), Egas Moniz School of Health & Science, Caparica, Almada, Portugal

**Keywords:** domestic violence, gender-based violence, neurocognitive deficits, cognitive functions, executive dysfunction, neuropsychological assessment

## Abstract

Intimate partner violence (IPV) significantly affects victims’ physical and mental health. Neurocognitive impairments, particularly in executive functioning, are crucial for daily functionality. However, no reviews or meta-analyses have focused on executive function (EF) alterations or the associated mechanisms contributing to a better understanding. This study aimed to synthesize and analyze differences in EF performance between women victims of IPV and non-victims, while also exploring potential mechanisms underlying these specific impairments. A systematic review and meta-analysis was conducted in accordance with the Preferred Reporting Items for Systematic reviews and Meta-Analyses guidelines. Searches were performed in the PubMed, Web of Science Core Collection, PsycINFO, and Scopus databases. A random-effects model was employed to calculate pooled effect sizes. A total of 22 studies published between 2002 and 2023 met the inclusion criteria and were included, comprising 1,425 women victims of IPV and non-victims. The meta-analysis revealed moderate to large effects on cognitive flexibility, inhibition, and semantic verbal fluency domains, with IPV victims showing lower performance. Working memory’s effect was non-significant. The qualitative synthesis demonstrated poorer phonological verbal fluency, nonverbal fluency, planning, reasoning, decision-making, and working memory. This synthesis also outlined differences observed among women experiencing physical, sexual, and/or psychological abuse, regardless of posttraumatic stress disorder or brain injury (BI). This systematic review and meta-analysis identified specific EF alterations in IPV victims, which may result from BI, psychopathology, or adverse childhood experiences, with their individual contributions requiring exploration in future studies. These findings can inform personalized interventions and decision-making within a legal context.

## Introduction

Intimate partner violence (IPV) is defined as “any behavior within an intimate relationship that causes physical, psychological, or sexual harm to those in the relationship” (Krug et al., 2002, p. 89). This includes physical aggression, psychological abuse, sexual coercion, and controlling behavior. IPV is recognized as both a human rights issue and a public health problem (Krug et al., 2002). Global estimates suggest that around 26% of women have experienced physical and/or sexual IPV, and 32.8% psychological IPV, though actual rates may be higher due to underreporting linked to stigma, methodological limitations, and cultural context (Sardinha et al., 2022; White et al., 2024).

IPV is associated with physical injuries, particularly to the head and neck (Kwako et al., 2011; Valera et al., 2022), which may result in traumatic brain injury (TBI) (Haag et al., 2022), including mild TBI (mTBI), a common yet often underreported consequence (Campbell et al., 2018). Chronic exposure to stress is also widespread (Daugherty et al., 2024; Kwako et al., 2011), leading to long-term mental health problems such as posttraumatic stress disorder (PTSD), depression, anxiety, self-harm, and eating disorders (Goldberg et al., 2021; White et al., 2024). PTSD is especially prevalent among IPV victims (Nathanson et al., 2012; White et al., 2024), with rates ranging from 51% to 75% (Nathanson et al., 2012). Complex PTSD (C-PTSD) also appears to be highly prevalent among IPV victims (Fernández-Fillol et al., 2021). This disorder encompasses a broader range of symptoms than PTSD, as it pertains to multiple, chronic, or repeated traumas, often occurring within interpersonal relationships beginning in childhood (Brewin et al., 2017). However, research on C-PTSD in IPV victims remains scarce.

Neurocognitive alterations among IPV victims have been consistently reported across several domains, including memory (Molinares et al., 2023; Raskin et al., 2023; Valera et al., 2022; Valera & Berenbaum, 2003), executive function (EF) (e.g., Daugherty et al., 2018; Raskin et al., 2023; Twamley et al., 2009), attention (Daugherty et al., 2018; Stein et al., 2002), processing speed (Aupperle et al., 2012; Meneses et al., 2023; Stein et al., 2002; Twamley et al., 2009), and visuoconstruction (Stein et al., 2002). These alterations may stem from mechanisms such as hypoxic/anoxic brain injury (BI) (Daugherty et al., 2018; Valera et al., 2022), TBI, psychopathology, adverse childhood experiences (ACEs) (Daugherty et al., 2024; Likitlersuang et al., 2023), and cortisol dysregulation (Daugherty et al., 2024). Brain imaging studies have corroborated structural and functional impairments in areas implicated in cognition and emotion regulation (Likitlersuang et al., 2023). Despite these findings, the mechanisms underlying the neurocognitive alterations in women victims of IPV are not fully understood (Daugherty et al., 2024).

Among these functions, EF – which involves the regulation of thought, action, and emotion (Diamond, 2013) – is essential for daily functioning. Core components include inhibition, working memory, and cognitive flexibility, which underpin higher-order skills such as planning, reasoning, decision-making, and verbal fluency (Amunts et al., 2020; Diamond, 2013). These functions also support emotion regulation and adaptive responses in social and occupational contexts (Zelazo, 2020). EF alterations may impact victims’ ability to leave abusive situations or access support, with evidence linking poorer EF performance to reduced ability to obtain material resources and increased risk of revictimization (Lee & DePrince, 2017).

While prior reviews have addressed general neurocognitive alterations in IPV victims (Daugherty et al., 2024; Kwako et al., 2011), only one meta-analysis has been conducted, without focusing specifically on executive functioning (García-Rueda & Jenaro, 2020). This systematic review and meta-analysis aims to: (a) synthesize evidence on EF performance in women victims of IPV; (b) explore potential mechanisms underlying cognitive impairments (e.g., PTSD, BI); and (c) identify research gaps to guide future studies.

## Method

A systematic review and meta-analysis was conducted following the Preferred Reporting Items for Systematic reviews and Meta-Analyses (PRISMA) guidelines (Page et al., 2021), and was registered in PROSPERO (registration number CRD42024574711). The PRISMA checklist is available in Table S1.

### Eligibility Criteria

Studies were included if they: (a) sampled females and/or individuals identifying as women; (b) included participants with current or past IPV exposure; (c) assessed EF with standardized neuropsychological tests; (d) used observational or experimental designs; and (e) were published in peer-reviewed and indexed journals. Exclusion criteria comprised: (a) participants under 18 (unless analyzed separately); (b) male-only or mixed-gender samples without disaggregated data; (c) exclusive focus on populations with unrelated neurological, psychiatric, or developmental disorders (but permeable to PTSD, C-PTSD, anxiety, depression, or personality disorders); (d) EF assessed solely by self-report; (e) reviews, meta-analyses, case series/reports; or (f) articles not in English, Spanish, French, or Portuguese.

### Data Sources and Search Strategy

The literature search comprised: (a) a search across four electronic databases; (b) forward and backward citation tracking, including previous relevant reviews; and (c) a manual search on Google Scholar to identify articles not retrieved by the previous procedures. The databases searched were PubMed (NHL), Web of Science Core Collection (Clarivate), PsycINFO (EBSCO), and Scopus (Elsevier). Initial searches were conducted on July 2, 2024, with results updated on November 15, 2024.

A search equation was developed using terms related to IPV and neurocognition, including subject headings and synonymous adapted for databases without controlled vocabulary. Relevant reviews (García-Rueda & Jenaro, 2020; Kwako et al., 2011) were consulted to ensure comprehensive term coverage. All terms were derived from the research question – “What are the executive function correlates of women victims of intimate partner violence?” – formulated using the PEO framework (Hosseini et al., 2024), which includes a “Study design (S)” component (see Table 1). Truncation (e.g., “neurocogniti*”) was used to capture term variations and adapted to each database. Searches were limited to the “Title or Abstract” fields, with no additional filters applied. Full search strategies per database are available in Table S2.

**Table 1.** PEOS Framework, Criteria, and Terms Applied in the Systematic Review Search.

PEOS	Criteria	Search Terms
Population (P)	Women/females	Female* OR woman OR women
Exposition (E)	Current or past experience of IPV	“Intimate partner violence” OR “intimate partner abuse” OR “domestic violence” OR “dating violence”
Outcomes (O)	Performance in standardized executive function tests	“Executive dysfunction*” OR “executive function*” OR “executive impairment*” OR “executive deficit*” OR “executive control” OR neuropsycholog* OR neurocogniti* OR cogniti* OR neurolog* OR neural
Study design (S)	Experimental or observational studies, excluding case series and case reports	NOT (review OR meta-analysis OR “case study” OR “case report” OR “case series”)

*Note*. IPV = Intimate partner violence.

### Selection Process

Records were imported into Zotero (Zotero, n.d.) for deduplication. The software identified potential duplicates by comparing the title, DOI, ISBN, publication year, and author(s), which were manually verified by SFN through cross-checking titles and authors before merging. The curated dataset was subsequently exported to Rayyan (Ouzzani et al., 2016) for screening and decision tracking. Two reviewers (SFN and MB) independently screened titles, abstracts, and full texts, blinded to each other’s decisions but not to study metadata. Discrepancies (98% agreement in abstract screening; 84.4% in full-text) were resolved through consensus or, when needed, consultation with senior reviewers (SF and RP). Senior reviewers were involved in four full-text cases reporting only descriptive data or focusing solely on brain activation without EF performance analysis.

### Data Extraction and Synthesis

Two reviewers (SFN and MB) independently extracted data from the included studies, as recommended by the Cochrane guidelines, to minimize errors during data extraction (Li et al., 2024). Data were extracted on author(s) and year of publication, country of origin, study design, participant characteristics, IPV definition and assessment, prevalence of BI and mental health outcomes, EF domains and tests used, and key findings. Relevant statistical data, including effect estimates, confidence intervals (CIs), test statistics, and *p*-values, were extracted for findings comparing EF performance between IPV victims and non-victims. When available, subgroup results (e.g., by PTSD, BI, or abuse type) were also retrieved. For cohort studies, descriptive results for IPV victims were collected at baseline, when data were available. For studies that analyzed different subgroups collectively (e.g., those including participants under 18 years and mixed sex/gender samples), descriptive results were specifically gathered for the subgroups of interest.

Authors were contacted for clarification when needed, and discrepancies were resolved with senior researchers (SF and RP). The percentage agreement between reviewers for data extraction was not calculated. Following the meta-analysis, a narrative synthesis was conducted.

### Risk of Bias and Quality Assessment

All studies were appraised using the Joanna Briggs Institute (JBI) Critical Appraisal Checklist for Analytical Cross-Sectional Studies (Moola et al., 2020), given the predominance of cross-sectional designs. A minority of cohort studies were also included, but assessed at a single time point to minimize bias. The checklist comprises eight questions that examine methodological quality and the extent to which potential biases are addressed in a study’s design, conduct, and analysis (detailed in Figure 4). Each question could be answered with “Yes”, “No”, “Unclear”, or “Not/Applicable”, with “Unclear” used when the article did not explicitly address the criterion.

The scoring system was defined in advance and agreed upon by all reviewers, as recommended by the JBI guidelines (Aromataris et al., 2024). In line with the criteria used in other systematic reviews (Franco et al., 2020; Payedimarri et al., 2022), the following thresholds were adopted: (a) scores above 70% “Yes” indicated low risk of bias; (b) 50%–69% indicated moderate risk; and (c) below 50% indicated high risk. Each article was independently assessed by two reviewers (SFN and MB), and any disagreements (73.9% agreement) were resolved through discussion with senior researchers (SF and RP). No articles were excluded based on the overall appraisal; however, those with a high risk of bias were interpreted with caution, and their limitations were addressed. The risk of bias plot was generated using the Robvis tool (McGuinness & Higgins, 2021).

### Statistical Synthesis Methods

A meta-analysis of EF performance was conducted for domains with similar data from at least three studies comparing IPV victims and non-victims (Myung, 2023). All analyses were performed using the Software Jamovi (The jamovi project, 2024).

A random-effects model was used to calculate pooled effect sizes, as it better accounts for uncertainty arising from heterogeneity among studies (Dettori et al., 2022). Due to missing information and variability in the reported analyses, means, standard deviations, and group sizes were extracted to compute the standardized mean difference (*SMD*), or Hedges’ *g*, as the effect size measure. Effects were considered significant when the 95% CI did not include zero. Forest plots with effect sizes and CIs are presented.

Heterogeneity was assessed using Cochran’s *Q*-test and *I*^2^ (low heterogeneity ≤ 25%, moderate ≈ 50%, high ≥ 75%) (Higgins et al., 2003), and Tau^2^ via Restricted Maximum Likelihood. Sensitivity analyses were conducted to assess the robustness and reliability of the results by including studies using different assessment instruments. The quality assessment scores were also analyzed as a potential source of methodological heterogeneity. Publication bias was evaluated using funnel plots and Egger’s regression test (β). Forest and funnel plots were generated using Jamovi (The jamovi project, 2024).

## Results

Following the search in four databases and other sources, and after screening, 22 studies were included. The PRISMA flow diagram detailing the number of studies at each screening phase is shown in Figure 1. Key information from each study is summarized in Table S3.

**Figure 1.**
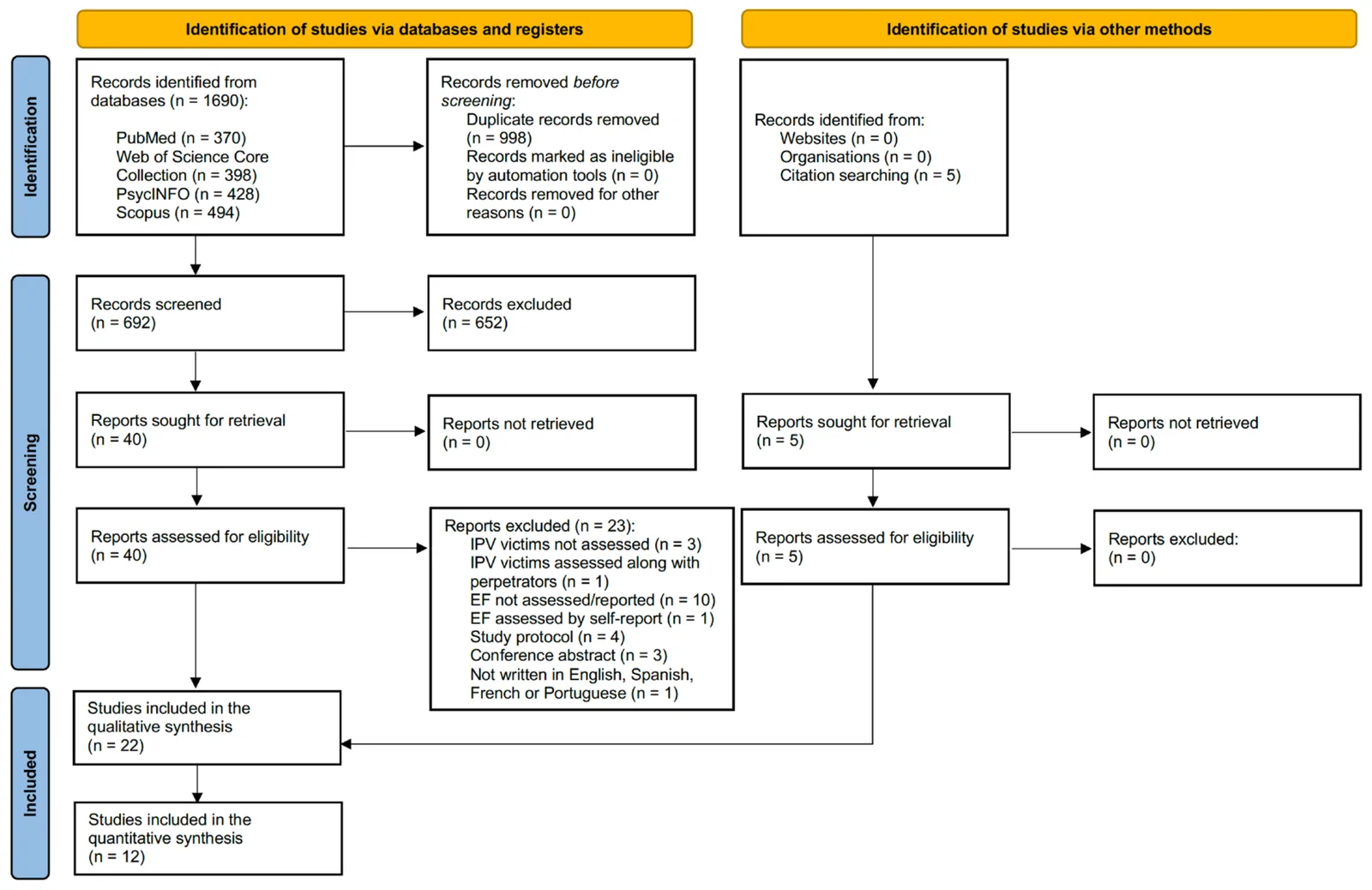
PRISMA 2020 flow diagram for systematic reviews. *Note*. IPV = Intimate partner violence; EF = Executive function.

### Study Setting and Design

The included studies were published between 2002 and 2023, with 40.91% conducted in the last 5 years. Most were from the USA (63.63%), followed by Spain (9.09%), Canada, Colombia, Ecuador, Mexico, Poland, and the Republic of China (each 4.55%). Using Grimes and Schulz’s (2002) taxonomy to classify design, most studies were cross-sectional (86.36%), with the remainder being cohort studies (13.64%).

### Sample Characteristics

Sample sizes ranged from eight to 236 women, totaling 1,425 participants – 1,091 IPV victims and 334 non-victims as controls. Victims were recruited mainly from IPV support organizations (68.18%), the general community (45.45%), shelters (31.81%), hospitals (13.64%), psychology/psychiatry services (9.09%), support groups (4.55%), and community coordinated programs (4.55%). Some studies used multiple sources. One study included participants as young as 17 years old (Dabkowska, 2007), but only descriptive data from adults were considered. Another study included male IPV victims (Williams et al., 2017), whose data were excluded.

### IPV Identification

IPV was most often defined as physical, psychological, and/or sexual abuse (31.81%), with other studies focusing on physical abuse alone (27.27%), physical and/or sexual abuse (13.64%), or physical and/or psychological abuse (9.09%). Nearly 18.18% did not specify IPV type.

Half of the studies used validated self-report tools, mostly the Revised Conflict Tactics Scale (CTS2), followed by the Conflict Tactics Scale (CTS), the Severity of Violence Against Women Scale, the Composite Abuse Scale Revised – Short Form, the Women’s Experiences with Battering, and a survey based on the Technical Standard for Comprehensive Attention to Gender Violence. The remaining studies did not use validated tools, with many of them recruiting participants through IPV-related organizations.

Relationship status was unspecified in most studies (50.09%). Some assessed recent IPV (last seven days to one year), others assessed IPV across the lifespan, or occurring 25 to 205 months earlier. About 27.27% required IPV to have ended before enrollment, and 13.64% included both current and past IPV, often within a 1-year timeframe. Few (13.64%) reported relationship duration, ranging from one to 240 months.

### BI Assessment

Most studies (77.27%) addressed BI, though assessment methods and criteria varied. Nearly 41% excluded participants with a history of moderate to severe BI, often defined by loss of consciousness >10 min, BI requiring hospitalization, or BI with cognitive dysfunction. In contrast, 36.36% included women with a history of BI, mostly IPV-related (BI, TBI, mTBI, or strangulation). A comprehensive overview of the BI assessment methods applied is provided in Table S4. BI prevalence ranged from 29% to 100% (this last percentage refers to studies where a history of BI was an inclusion criterion; see Table S3 for prevalence data reported in each study).

### Mental Health Outcomes Assessment

PTSD (77.27%), depression (77.27%), and anxiety (59.09%) were the most frequently assessed mental health conditions. Assessment criteria varied: some studies included only IPV victims meeting full or partial PTSD diagnosis, while others included participants regardless of diagnosis, often analyzing symptom severity dimensionally. Some divided IPV victims into groups with current and/or lifetime PTSD diagnosis and those without. A comprehensive overview of the PTSD assessment methods applied is provided in Table S4. IPV-related PTSD prevalence ranged from 25.9% to 100% (this last percentage refers to studies where a PTSD diagnosis was an inclusion criterion). Depression and anxiety symptoms were analyzed dimensionally, with moderate/severe prevalence ranging from 23% to 71% for depression and 24% to 80% for anxiety. Only one study excluded participants with PTSD, depression, or anxiety (Daugherty et al., 2018). Prevalence data for PTSD, depression, and anxiety are provided in Table S3.

Substance abuse was also assessed in 22.73% of the studies, but was excluded in over half (54.55%). Less frequently assessed outcomes included quality of life, life satisfaction, disability, sleep quality, personality disorders, dissociation, and worry.

The qualitative synthesis focuses on EF performance in relation to PTSD, depression, and anxiety, as these were the most assessed and prevalent mental health outcomes among IPV victims (White et al., 2024).

### ACEs Assessment

ACEs were considered in 22.72% of studies. Only one examined their association with EF (Twamley et al., 2009). Some included ACEs as a covariate (Valera & Kucyi, 2016; Valera et al., 2019, 2022), while one explored the association with brain volume (Fennema-Notestine et al., 2002). Prevalence data were mostly unreported (see Table S3), except one study reporting that four out of ten women with PTSD had experienced ACEs (Aupperle et al., 2016).

### EF Assessment

EFs assessed varied across studies, as did the neuropsychological tests used for each domain. Cognitive flexibility was most frequently evaluated (86.36%), followed by inhibition (45.45%), working memory (45.45%), verbal/nonverbal fluency (36.36%), planning (13.64%), reasoning (13.64%), and decision-making (4.55%). EF domains and their respective tests across all included studies are presented in Table S5, ordered by the most frequently used test for each domain, along with the corresponding frequencies. The terminology for EF varied widely across studies, and some assessed these domains without explicitly labeling them as such. To address this, terms were standardized for this review, with the original author terms specified in Table S3.

Some studies used multiple tests per domain and showed inconsistencies in classification. For instance, the Wisconsin Card Sorting Test (WCST) was identified as assessing both cognitive flexibility and abstract reasoning in one study (Aupperle et al., 2012), but was classified exclusively as a measure of reasoning in another (Twamley et al., 2009). To ensure consistency, WCST results were categorized under reasoning in this review. Similarly, the Trail Making Test – Part B (TMT-B) was considered to assess both cognitive flexibility and working memory in one study (Dabkowska, 2007), but since it is widely classified as a measure of cognitive flexibility in the other studies, its findings were reported under that domain. The Controlled Oral Word Association Test was used as a measure of language ability in one study (Stein et al., 2002), but because it includes semantic and phonological verbal fluency subtests and was identified as verbal fluency in another study (Raskin et al., 2023), results were presented in the verbal fluency domain. A table summarizing the original and reclassified EF or cognitive domains of neuropsychological tests, along with the corresponding justifications, is presented in Table S6.

### Quantitative Synthesis

Twelve studies were included in the meta-analysis: three assessed cognitive flexibility, three working memory, three inhibition, and three semantic verbal fluency. Only the most frequently used test per domain – applied in at least three studies to compare EF performance between IPV victims and non-victims – was included in the analysis. These criteria were set to ensure comparability across studies, given the variability in outcome measures and scoring systems. The studies selected for inclusion in the meta-analysis, along with the respective tests and statistical data, are presented in Table S7. Subgroup analyses by PTSD diagnosis or moderate/severe BI were not feasible due to insufficient studies (fewer than two per subgroup), as suggested by Myung (2023).

The meta-analysis revealed large, significant overall estimated effects for differences in cognitive flexibility (*SMD* = 0.80, 95% CI [0.48, 1.11]; *Z* = 4.91, *p* < .001) and inhibition (*SMD* = −1.05, 95% CI [−1.79, −0.31]; *Z* = −2.80, *p* = .005) between IPV victims and non-victims, with victims exhibiting worse performance. The CI amplitude for cognitive flexibility – the difference between upper and lower CI limits – was relatively low (0.63) and smaller than the effect size, indicating greater precision. In contrast, inhibition had a wider CI amplitude (1.48) exceeding the effect size, suggesting lower precision. Semantic verbal fluency showed a moderate, significant effect (*SMD* = −0.45, 95% CI [−0.72, −0.18]; *Z* = −3.22, *p* = .001), also worse in victims. Its CI amplitude (0.54) was slightly larger than the effect size but still indicated good precision. These results are illustrated in the forest plots (Figure 2). Raskin et al. (2023) contributed most weight to the cognitive flexibility, inhibition, and semantic verbal fluency models.

**Figure 2.**
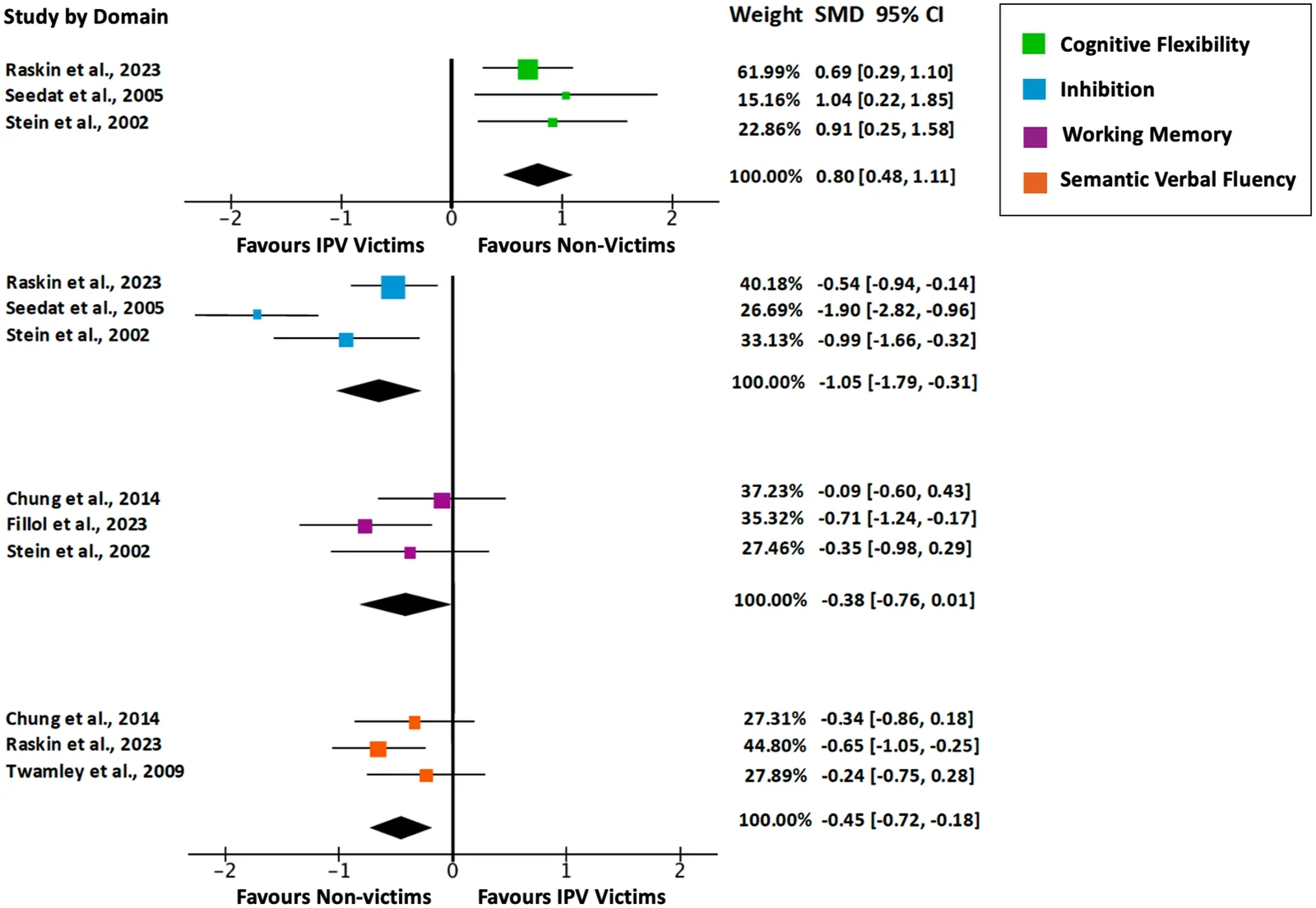
Forest plots for cognitive flexibility, inhibition, working memory, and semantic verbal fluency domains, including studies’ weights, standardized mean differences at 95% confidence interval, and values for overall random effects model. *Note.* IPV = Intimate partner violence; SMD = Standardized Mean Difference; CI = Confidence Interval.

Regarding working memory, the analysis showed a weak, negative, and non-significant overall effect (*SMD* = −0.38, 95% CI [−0.76, 0.01]; *Z* = −1.93, *p* = .054) between IPV victims and non-victims (Figure 2). The wide CI (0.75) exceeded the overall estimate, indicating low precision. Chung et al. (2014) contributed the most weight to this model.

Low heterogeneity was observed for cognitive flexibility (*Q* = 0.71, *p* = .701; *I*^2^ = 0%; Tau^2^ = 0) and semantic verbal fluency (*Q* = 1.81, *p* = .405; *I*^2^ = 2.4%; Tau^2^ = .001). Moderate heterogeneity was obtained for studies analyzing working memory (*Q* = 2.69, *p* = .260; *I*^2^ = 29.42%; Tau^2^ = .034) and inhibition (*Q* = 7.38, *p* = .025; *I*^2^ = 74.85%; Tau^2^ = .312), with inhibition nearing high heterogeneity.

Egger’s regression test indicated no publication bias for cognitive flexibility (β = 0.84, *p* = .399), working memory (β = –0.08, *p* = .934), and semantic verbal fluency (β = 1.31, *p* = .192), supported by symmetric funnel plots (Figure 3). For inhibition, Egger’s regression (β = –2.65, *p* = .008) and funnel plot asymmetry (Figure 3) suggested publication bias.

**Figure 3.**
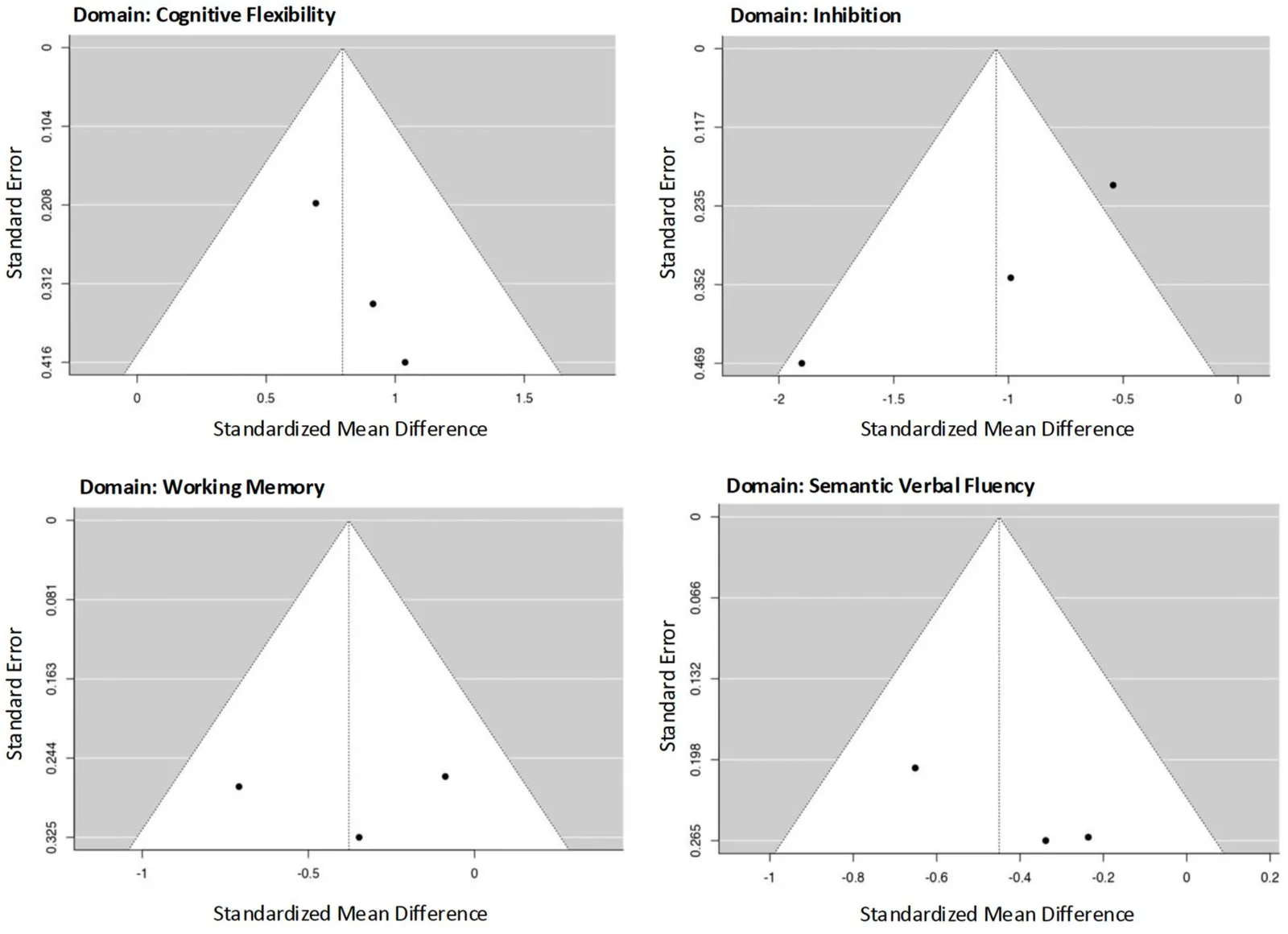
Funnel plots of effect sizes for cognitive flexibility, inhibition, working memory, and semantic verbal fluency domains.

A sensitivity analysis was performed, including the original studies and additional studies comparing IPV victims and non-victims within the EF domains meta-analyzed. These studies considered the various neuropsychological tests used beyond those selected for the original analysis. The additional studies, along with their respective tests and statistical data, are listed in Table S8.

Within the sensitivity analysis, the overall estimated effect for differences between groups in cognitive flexibility (*k* = 7) decreased from large to moderate but remained significant (*SMD* = 0.57, 95% CI [0.36, 0.78]; *Z* = 5.35, *p* < .001), with low heterogeneity (*Q* = 7.61, *p* = .268; *I*^2^ = 13.53%; Tau^2^ = 0.01). Semantic verbal fluency (*k* = 4) retained a moderate and significant effect (*SMD* = −0.47, 95% CI [−0.72, −0.22]; *Z* = −3.69, *p* < .001), also with a low heterogeneity (*Q* = 1.92, *p* = .589; *I*^2^ = 0%; Tau^2^ = 0).

The effect for working memory (*k* = 4) increased from weak and non-significant to moderate and significant (*SMD* = −0.45, 95% CI [−0.72, −0.17]; *Z* = −3.17, *p* = .002), with a heterogeneity decreasing from moderate to low (*Q* = 3.25, *p* = .354; *I*^2^ = 11.05%; Tau^2^ = .009). In contrast, the effect for inhibition (*k* = 6) decreased from large to moderate and lost significance (*SMD* = −0.48, 95% CI [−1.17, −0.22]; *Z* = −1.34, *p* = .181), with heterogeneity increasing from moderate to high (*Q* = 43.243, *p* < .001; *I*^2^ = 90.45%; Tau^2^ = .671).

### Qualitative Synthesis

Compared with non-victims, IPV victims presented lower performance across several EF domains, including cognitive flexibility, inhibition, working memory, verbal and nonverbal fluency, planning, reasoning, and decision-making (see Table S3). The qualitative synthesis below focuses only on domains with significant overall effects in the meta-analysis: cognitive flexibility, inhibition, and semantic verbal fluency, including studies from both the meta-analysis and others addressing these domains.

#### Cognitive Flexibility

Among eight studies comparing IPV victims and non-victims, results were inconsistent. About 37.5% reported significantly lower performance among IPV victims with PTSD (Aupperle et al., 2012) or regardless of PTSD diagnosis (Raskin et al., 2023; Seedat et al., 2005), and below-average performance relative to normative data was observed (Meneses et al., 2023). Half of the studies found no significant differences, regardless of whether the victims had PTSD (Aupperle et al., 2016; Chung et al., 2014; Twamley et al., 2009) or not (Daugherty et al., 2018). Only one study, including women with potential BI or TBI, found significant group differences but no effects of strangulation or loss of consciousness duration (Raskin et al., 2023). One study found mixed results, namely worse performance in victims with PTSD versus non-victims, but no difference between victims with and without PTSD (Stein et al., 2002).

It is relevant to note that both studies reporting significant and no significant differences in cognitive flexibility recruited IPV victims from diverse settings, including the general community, hospitals, psychiatric clinics, IPV services/agencies, and shelters.

Most studies found no correlations with PTSD symptom severity (Aupperle et al., 2012, 2016; Meneses et al., 2023; Seedat et al., 2005; Stein et al., 2002), though two reported associations with greater PTSD severity (Chung et al., 2014; Molinares et al., 2023).

Regarding BI severity, findings were mixed. Half reported worse performance linked to higher IPV-related BI severity (Molinares et al., 2023; Valera & Berenbaum, 2003), but these associations lost significance after controlling for PTSD symptom severity (Molinares et al., 2023; Valera & Berenbaum, 2003), socioeconomic status, and educational level (Molinares et al., 2023). Other studies found no correlation with BI severity (Valera & Kucyi, 2016) or strangulation-related alterations in consciousness (Valera et al., 2022).

Differences by abuse type were inconsistent. Lower performance was found in victims of psychological and physical abuse compared with psychological abuse only (Daugherty et al., 2018). Clinically significant alterations appeared in sexual violence victims but not in psychological or physical IPV victims (Meneses et al., 2023), although no differences were found between IPV and sexual assault victims (Raskin et al., 2023).

Neuroimaging studies reported associations between worse cognitive flexibility performance in IPV victims and reduced frontal cortical gray matter and smaller cranial vault volumes, with the latter associated with childhood physical abuse (Fennema-Notestine et al., 2002). Similarly, ACEs were associated with worse cognitive flexibility in another study (Twamley et al., 2009).

No significant correlations were found with depressive (Aupperle et al., 2012; Meneses et al., 2023; Seedat et al., 2005; Stein et al., 2002) or anxiety symptoms (Meneses et al., 2023; Seedat et al., 2005).

#### Inhibition

Among seven studies, more than half (57.14%) reported significantly lower inhibition performance in IPV victims, with some including victims with or without PTSD (Meneses et al., 2023; Raskin et al., 2023; Seedat et al., 2005; Stein et al., 2002), and a single study focused solely on victims without PTSD (Daugherty et al., 2018). All studies, except one (Raskin et al., 2023), excluded women with a history of possible BI or TBI. The remaining studies (42.86%), including IPV victims with PTSD and no history of moderate/severe BI, found no significant differences in inhibition performance (Aupperle et al., 2012, 2016; Twamley et al., 2009).

The studies that found significant differences in inhibition performance recruited IPV victims from shelters, support groups, hospitals, or specialized IPV services. In contrast, the studies that did not find significant differences recruited victims not only from hospitals, psychiatric clinics, and IPV-related services, but also from the general community.

No significant differences were found between victims with and without PTSD (Stein et al., 2002), nor correlations with PTSD severity (Aupperle et al., 2012, 2016; Meneses et al., 2023; Seedat et al., 2005; Stein et al., 2002).

No study examined inhibition performance in relation to BI severity except one, which found no effect of non-fatal strangulation or loss of consciousness duration (Raskin et al., 2023).

Studies exploring differences between types of abuse did not yield significant results (Daugherty et al., 2018; Meneses et al., 2023), although one study found poorer performance in victims of physical and psychological abuse compared with non-victims, but no differences between victims of psychological abuse alone and non-victims (Daugherty et al., 2018). No differences emerged between IPV victims and sexual assault victims, nor between sexual assault victims and non-victims (Raskin et al., 2023).

No correlations were found between inhibition performance and depressive (Aupperle et al., 2012; Meneses et al., 2023; Seedat et al., 2005; Stein et al., 2002; Twamley et al., 2009), anxiety symptoms (Meneses et al., 2023; Seedat et al., 2005), or ACEs (Twamley et al., 2009).

#### Semantic Verbal Fluency

Findings across four studies were inconsistent. One (25%) found poorer performance in IPV victims with or without PTSD (Raskin et al., 2023), and another reported worse performance compared with normative data (Meneses et al., 2023). Half of the studies found no significant differences between victims and non-victims, regardless of PTSD diagnosis (Chung et al., 2014; Twamley et al., 2009). One study reported mixed results, noting significant differences between victims without PTSD and non-victims, but no differences between victims with PTSD and non-victims (Stein et al., 2002).

Studies that found significant differences in semantic verbal fluency recruited IPV victims from shelters, support groups, and hospitals, whereas those that did not find significant differences recruited participants from both the general community and IPV-prevention services.

No differences were found between victims with and without PTSD (Chung et al., 2014), and no correlations with PTSD severity were reported (Meneses et al., 2023; Stein et al., 2002), except in one study (Chung et al., 2014).

No significant differences emerged by abuse type (Meneses et al., 2023) or between IPV and sexual assault victims (Raskin et al., 2023).

Only one study that included victims with possible BI or TBI reported poorer fluency performance in women experiencing non-fatal strangulation, without correlation to loss of consciousness duration (Raskin et al., 2023).

No correlations were found with depression (Meneses et al., 2023; Stein et al., 2002; Twamley et al., 2009), anxiety symptoms (Meneses et al., 2023), or ACEs (Twamley et al., 2009).

### Risk of Bias and Study Quality

Details regarding the risk of bias assessment for each study, based on the JBI Critical Appraisal Checklist for Analytical Cross-Sectional Studies (Moola et al., 2020), are presented in Figure 4. Overall, 23% of studies were rated high risk, 50% moderate, and 27% low risk of bias. The most frequent high-risk domain was “Was the exposure measured in a valid and reliable way?” due to most studies not using specific IPV assessment instruments. The domains “Were objective, standard criteria used for measurement of the condition?” and “Were the outcomes measured in a valid and reliable way?” were often rated “Unclear”, reflecting vague definitions of abuse type and duration, as well as whether IPV exposure was past or current. Lack of information on who administered neuropsychological tests also hindered the assessment of measurement validity and reliability.

**Figure 4.**
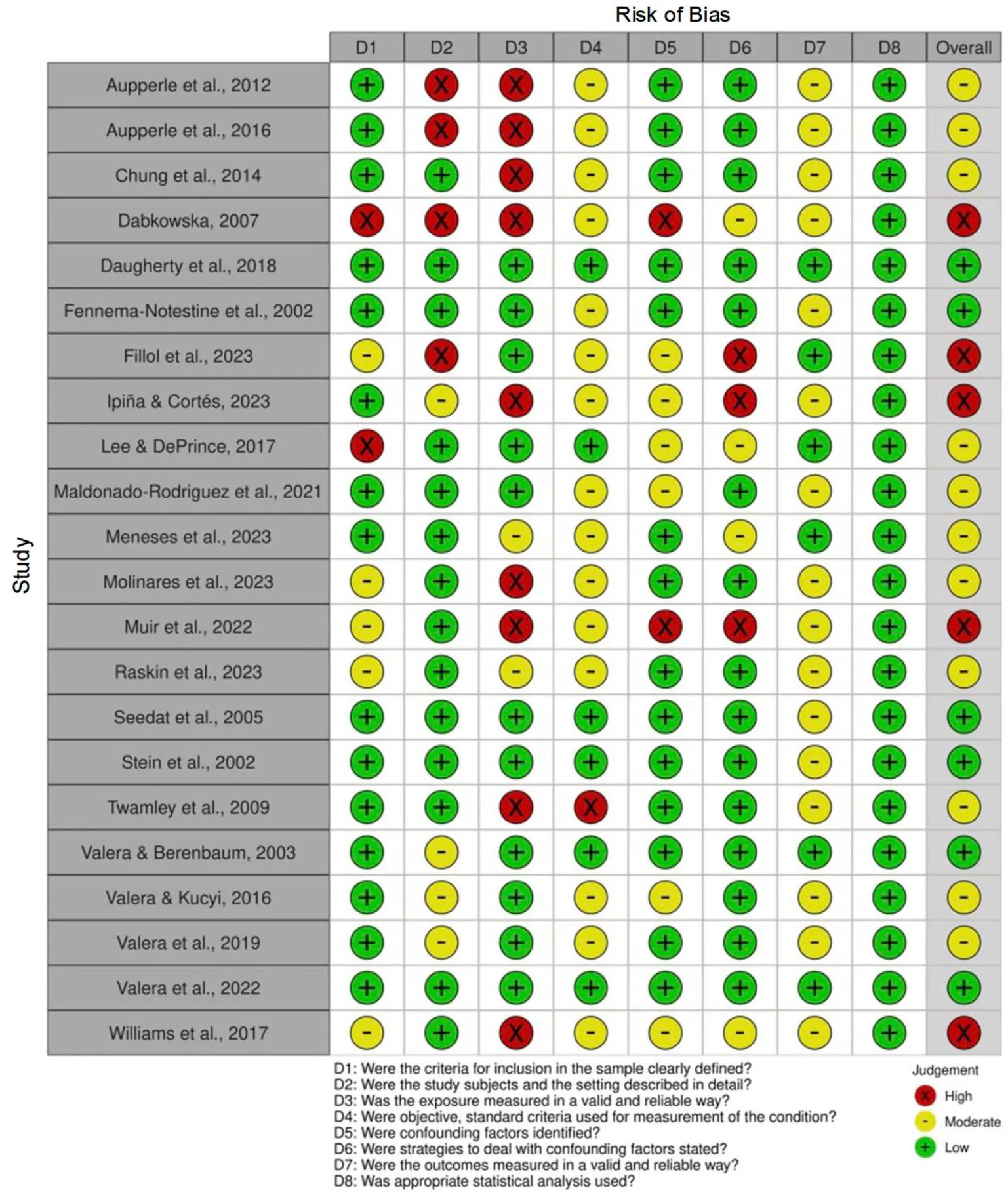
Summary of risk of bias assessment.

## Discussion

This systematic review and meta-analysis provide novel contributions to identifying and understanding executive functioning in women victims of IPV. A summary of key findings is presented in Table 2.

**Table 2.** Summary of Critical Findings.

• Women exposed to IPV showed poorer executive functioning compared with non-victims, particularly in cognitive flexibility, inhibition, and semantic verbal fluency, with moderate to large effect sizes observed in these domains.
• Alterations in other EF domains were identified in the qualitative synthesis, namely phonological verbal fluency, nonverbal fluency, planning, reasoning and decision-making.
• Working memory did not show significant differences between victims and non-victims in the meta-analysis, although qualitative results indicated possible deficits.
• Altered EF performance occurred regardless of PTSD diagnosis or BI, suggesting multifactorial etiological mechanisms; some studies did find that women with BI or PTSD had worse performance, but these associations were not consistent across the literature.
• Potential mechanisms contributing to EF alterations include TBI (especially mild TBI), PTSD and other psychopathologies (e.g., depression, anxiety), and ACEs, all of which may exert cumulative neurobiological effects on cognitive function.

*Note.* IPV = Intimate partner violence; EF = Executive function; PTSD = Posttraumatic stress disorder; TBI = Traumatic brain injury; ACEs = Adverse childhood experiences; BI = Brain injury.

The meta-analysis revealed moderate to large effects in cognitive flexibility, inhibition, and semantic verbal fluency, aligning with prior findings highlighting EF and memory as vulnerable domains in IPV victims (García-Rueda & Jenaro, 2020). The qualitative synthesis corroborated lower EF performance across multiple domains, consistent with earlier reviews on neuropsychological alterations in IPV victims (Daugherty et al., 2024; Kwako et al., 2011). EF alterations appear across physical, sexual, and psychological abuse, with cumulative abuse types linked to greater severity (Daugherty et al., 2018). This converges with evidence of IPV’s impact on mental (White et al., 2024) and physical health (Stubbs & Szoeke, 2022).

Overall, the sensitivity analysis supports the robustness of the effects obtained in the meta-analysis for EF differences between IPV victims and non-victims. For instance, the effects for cognitive flexibility and semantic verbal fluency remained significant after the inclusion of additional studies, with low heterogeneity, indicating consistent findings across studies. While semantic verbal fluency maintained a moderate effect, the effect for cognitive flexibility decreased from large to moderate. This reduction may reflect the use of different assessment methods with varying levels of difficulty or differences in sample characteristics; although most studies included women with PTSD, the additional studies did not involve participants with a history of moderate/severe BI.

Notably, working memory shifted from non-significant to a moderate and significant effect, suggesting greater robustness when more data are considered. In this case, the added study involved women exposed to both physical and psychological violence (Daugherty et al., 2018), whereas previous studies did not specify the type of violence (only reporting past psychological, physical, and/or sexual violence). While the additional study included women without PTSD or a history of moderate/severe BI, cumulative trauma exposure – potentially including unassessed mTBI – may have contributed to more pronounced alterations in working memory. In contrast, the effect for inhibition lost significance, with heterogeneity increasing to a high level. This suggests that the additional studies amplified inconsistencies, possibly due to differences in assessment methods or sample characteristics – most of which included participants with PTSD but without moderate/severe BI. Therefore, the effect for inhibition should be interpreted with caution.

The qualitative synthesis revealed some inconsistencies across studies comparing IPV victims and non-victims in cognitive flexibility, inhibition, and semantic verbal fluency. One potential explanation concerns the recruitment setting of IPV participants. Specifically, for cognitive flexibility, both significant and non-significant results were observed in studies that recruited participants from diverse sources – including the general community, hospitals, clinics, and IPV-related services. Recruitment from multiple settings makes it difficult to isolate the influence of any one context, since each may involve distinct profiles of abuse severity and psychopathology. For inhibition and semantic verbal fluency, a pattern emerged: significant differences appeared in studies recruiting from hospitals, clinics, or IPV-related services, while studies including IPV victims from the general community tended to report non-significant results. Although it is difficult to determine which setting had the greatest impact, it is possible that IPV victims from the general community experience less severe abuse or psychopathology, as they may not have sought clinical or specialized support. It may be hypothesized that cognitive flexibility alterations, unlike working memory and semantic verbal fluency, are observable among IPV victims across a wider range of settings, regardless of abuse severity or psychopathology. Nonetheless, it is relevant to acknowledge that some victims in the general community may still experience severe abuse or psychopathology but avoid seeking help due to stigma (White et al., 2024).

Moreover, two studies reporting no significant differences in cognitive flexibility and inhibition recruited victims through self-reported phone interviews (Aupperle et al., 2016; Twamley et al., 2009). The lack of a validated screening tool in these cases may have contributed to possible misclassification of IPV victims.

Worse EF performance was found in IPV victims regardless of BI history. Most studies excluded moderate/severe BI, indicating cognitive alterations are not solely due to BI from IPV (Daugherty et al., 2024). However, mTBI may have been present undetected, potentially affecting results (Naumenko et al., 2023).

Interestingly, Raskin et al. (2023) reported that 95% of IPV victims in their sample screened positive for possible BI, and 60% for possible TBI, based on a validated screening tool. This study presented significant EF differences between victims and non-victims and contributed the most weight to the meta-analytic models, possibly due to BI-related effects or the relatively large sample size. In addition to TBI, the study analyzed the impact of non-fatal strangulation on EF, finding no significant differences in cognitive flexibility or inhibition, although significant differences were observed in semantic verbal fluency between victims and non-victims. Similarly, Valera et al. (2022) reported no significant cognitive flexibility differences between IPV victims with and without strangulation-related alterations in consciousness, although significant differences were found for working memory. These findings suggest that strangulation may be more specifically associated with EF domains that are more dependent on memory processes, compared to other EF domains (Kavé & Sapir-Yogev, 2020). This hypothesis aligns with evidence indicating that the hippocampus – a region critical for memory – is particularly vulnerable to hypoxia (Lu et al., 2025), which can result from oxygen deprivation during strangulation. Notably, the studies on working memory included in the original meta-analysis either excluded participants with a history of BI or did not assess this variable, which may help explain the absence of a significant overall effect.

Even though IPV-related head and neck injuries may damage brain regions and neurotransmitter systems critical for cognition (McAllister, 2008), correlations between BI and EF were inconsistent. Indeed, a study reported that by 12 months postinjury, most individuals with moderate or severe TBI had regained daily independence, though some cognitive impairments may persist (McCrea et al., 2021). Most mTBI cases also show cognitive recovery, with fewer lasting effects (Rabinowitz & Levin, 2014). Some null findings may reflect assessments conducted beyond the acute injury phase.

Lower EF performance was observed in IPV victims with and without PTSD, suggesting broader underlying mechanisms (Daugherty et al., 2024). Most studies included both women with and without PTSD, making it difficult to isolate effects. However, cognitive flexibility exhibited a distinct pattern, since significant impairments were absent in samples of IPV victims without PTSD (Aupperle et al., 2012; Raskin et al., 2023; Seedat et al., 2005; Stein et al., 2002), and some non-significant findings were reported specifically for this group (Daugherty et al., 2018; Stein et al., 2002). Supporting this, a study in IPV victims found that PTSD severity was negatively correlated with dorsolateral prefrontal cortex activation during the anticipation of negative images, with higher activation linked to better cognitive flexibility performance (Aupperle et al., 2012). Also, amygdala and insula activation were positively correlated with PTSD severity during the anticipation of negative emotional images. These findings suggest that hypervigilance in PTSD contributes to difficulties in disengaging and reorienting from internal emotional states and traumatic memories to external and novel stimuli (Aupperle et al., 2012).

Controversially, many studies failed to find significant differences in EF performance between IPV victims with and without PTSD, nor correlations with PTSD symptom severity, Some authors attribute these findings to small sample sizes (Stein et al., 2002), while others suggest that cognitive alterations may be linked more to the adverse experience itself than to PTSD symptoms (Chung et al., 2014; Stein et al., 2002). Cognitive flexibility was also uniquely correlated with ACEs (Twamley et al., 2009). Accordingly, ACEs are related to dysregulated stress responses (Murphy et al., 2022) and may lead to structural and functional changes in prefrontal and limbic regions involved in emotion regulation and cognitive control (McLaughlin et al., 2014). Indeed, in IPV victims with PTSD, ACEs are linked to abnormal brain activation and gray matter volume in executive areas (Fonzo et al., 2013).

No study has yet explored whether ACEs contribute to IPV victimization. Longitudinal data show 24.5% of IPV victims self-report childhood maltreatment (Kisely et al., 2024), a known risk factor for poor mental health (Murphy et al., 2022) and IPV victimization (Spencer et al., 2019). Despite converging evidence, the lack of longitudinal studies limits causal inferences regarding ACEs, EF alterations, and IPV victimization (Daugherty et al., 2024; Stein et al., 2002).

### Limitations and Future Research Directions

The present review and meta-analysis has several limitations that should be considered when interpreting the findings, predominantly stemming from methodological heterogeneity across included studies. Although most were rated moderate or high quality, the limited number of studies precluded excluding articles based solely on quality.

Heterogeneity was evident in IPV identification, with distinct definitions, assessment methods, and recruitment sites, possibly leading to underreporting (White et al., 2024). Many studies did not specify whether IPV was current or past, nor controlled for abuse duration or time since relationship end. Despite the recruitment of IPV victims from shelters or specialized services in some studies, others that rely on community samples use severity instruments without specific thresholds for IPV identification. Future research should address these factors and compare EF profiles between women in ongoing versus ended abusive relationships across diverse backgrounds. The establishment of clearer and standardized criteria for defining IPV victimization is also recommended.

As highlighted in a previous meta-analysis (García-Rueda & Jenaro, 2020), neuropsychological tests and scoring systems varied widely. Decisions made to standardize the results could have led to the exclusion of relevant findings. Regarding the reclassification of EF domains for certain neuropsychological tests, the WCST was categorized as a measure of reasoning in the present review for consistency across studies. However, as this test is also widely recognized as assessing cognitive flexibility, this standardization may have influenced the meta-analytic results and their interpretation. In accordance, the original meta-analysis focused on the most frequently used test within each EF domain among studies comparing IPV victims and non-victims, thereby excluding relevant data from other psychometrically valid neuropsychological tests; the sensitivity analysis partly addressed this limitation. However, this approach was intended to enhance the clarity and interpretability of the evidence.

Although the included studies did not consistently specify whether the instruments used were self-administered or clinician-administered, all involved cognitive performance tasks that inherently require the direct involvement of an examiner – such as for providing standardized instructions, monitoring timing, observing participant behavior, and scoring responses. The exceptions were a few studies that employed computerized batteries (Fillol et al., 2023; Molinares et al., 2023) or specific computerized tests (Daugherty et al., 2018; Muir et al., 2022). This lack of explicit reporting, combined with the reliance on clinician-administered assessments, may introduce a potential source of bias, as performance outcomes could be influenced by the examiner’s interpretation or the interpersonal dynamics established during the assessment process. Furthermore, some studies did not report the qualifications or experience of the individuals administering the neuropsychological tests, raising reliability concerns. Variability in examiner training and credentials across studies may further constitute a potential source of bias. These limitations should be considered when interpreting the findings of the present review.

Studies relying solely on self-report EF assessments were excluded to enhance comparability, though integrating victim perspectives could enrich understanding (Daugherty et al., 2022). To date, no studies have employed a mixed-method approach.

BI assessment methods also varied, often relying on self-report, which may underestimate prevalence (Raskin et al., 2023). Additionally, while some studies used standardized screening tools, others did not specify their assessment methods or relied solely on participants’ self-reports. The lack of neuroimaging-confirmed diagnoses is a limitation, as it restricts the ability to draw robust conclusions and, when available, could provide valuable information about the location of potential brain lesions. These factors, combined with the varying operational definitions of BI, limit comparability across studies. Also, most studies did not account for mTBI, a potential confounder. Future studies should use validated screening tools for mild to severe TBI and strangulation, considering injury location and time since injury to clarify cognitive impact and mechanisms.

Most studies assessed current PTSD but not lifetime diagnosis, limiting understanding of whether EF alterations relate specifically to IPV-related PTSD or cumulative trauma. Furthermore, no studies have yet examined EF in IPV victims with C-PTSD. Future studies should compare EF among IPV victims with PTSD versus C-PTSD, and between victims and non-victims with C-PTSD, to clarify contributors to cognitive alterations.

Premorbid intelligence was rarely assessed, potentially contributing to cognitive profile inconsistencies. In line with this, three longitudinal studies were included in the review, although only cross-sectional results were considered to ensure homogeneity and data comparability. One study assessed EF performance prior to an IPV incident and compared it with post-incident performance (Williams et al., 2017). In the other two, women were assessed after an IPV incident, with EF performance either followed up over time (Muir et al., 2022) or analyzed in relation to other variables (Lee & DePrince, 2017). Longitudinal designs are crucial to distinguish pre-existing EF alterations from IPV consequences and to explore the role of ACEs and prior mental health. Nevertheless, following a group of women at risk of IPV would raise ethical concerns, as it would not be feasible to allow them to engage in an abusive relationship (Daugherty et al., 2024). However, the role of EF in daily functioning and their relationship with risk factors for IPV victimization (e.g., ACEs, lack of material resources) justifies exploring EF as a predictor – beyond being a consequence – through alternative research designs.

Gray literature was excluded since it is not subjected to a peer-review process, which is considered a reliable proxy for quality (Adams et al., 2017). Moreover, it tends to increase the heterogeneity of evidence retrieval and synthesis. The selection was also restricted to articles in languages understood by the authors. These aspects may have led to the exclusion of relevant literature, whose inclusion could help reduce publication bias (Adams et al., 2017) and contribute to a more culturally comprehensive review.

While the percentage of agreement exceeded 80% during the screening process – supporting the robustness of the results – the agreement between reviewers for the quality assessment was 73.9%, reflecting moderate interrater reliability. This level is considered acceptable, and senior reviewers contributed to resolving disagreements. However, it falls slightly below the commonly recommended threshold of 80% and may indicate some subjectivity in the evaluation process. Additionally, interrater agreement for data extraction was not calculated, representing a limitation that should be considered when interpreting the findings.

Regarding the quality assessment, it is important to note that the threshold was defined based on previous reviews, given the absence of standardized criteria. However, those reviews examined populations other than IPV victims, and to our knowledge, no prior review has established thresholds for the JBI Checklist specifically within IPV samples. This represents a limitation, as such thresholds – while useful – are ultimately arbitrary and may not fully capture the specificities of IPV-related research, including variability in how IPV exposure is identified and classified.

The meta-analysis was constrained by the small number of studies per EF domain and methodological weaknesses, including moderate to high bias risk, compromising the robustness of the estimated effects. The ability to carry out subgroup analyses was also restricted by the number of studies, limiting the exploration of mechanisms behind EF differences. Consequently, this review qualitatively explores factors such as PTSD, BI, and other psychopathologies (e.g., depression, anxiety) as potential mechanisms underlying EF alterations. However, when comparing IPV victims with non-victims, additional comorbidities not consistently reported or controlled for may also contribute to the observed differences, potentially influencing the interpretation of the findings. In addition, including studies with a high risk of bias may have contributed to heterogeneity and could justify a meta-regression if more studies are available. As often emphasized, studies with larger sample sizes are necessary to capture more subtle differences between groups. Bayesian methods may be applied to address estimation imprecision arising from a small number of studies (van Aert & Mulder, 2022). Future studies could also implement stratified analysis by examining comorbidity patterns, abuse typology, and diversity-related variables to provide a deeper understanding and address the limitations identified in the current research.

Focusing primarily on women limits generalizability. Research on men and gender-diverse LGBTQIA2S+ populations is scarce despite high stigmatization (Gram et al., 2024). There may be specific cognitive profiles in these populations that warrant investigation to develop tailored psychological interventions and cognitive rehabilitation programs.

### Clinical and Legal Implications

Several implications for both practice and policy can be underscored. Clinicians may incorporate routine neuropsychological assessments using validated batteries for IPV victims, helping to guide rehabilitation interventions (Daugherty et al., 2018). The possible role of EF in victimization may be considered even among non-victimized women at risk of IPV. Despite the lack of studies in this field, the early identification of EF alterations may contribute to the development of prevention programs.

In a legal context, recognizing these impairments and their impact can inform court decisions (Daugherty et al., 2018). This recognition can facilitate both the allocation of adequate social support and improved access to financial compensation for IPV-related sequels, thereby increasing the autonomy and well-being of victims. Such support must address the consequences of physical, sexual, and psychological violence since all are associated with significant alterations.

Policies improving access to education, healthcare, and employment may enhance women’s independence, enabling them to challenge discriminatory gender norms and thereby contributing to IPV prevention (Ma et al., 2023).

Implications of the review for practice, policy, and research are presented in Table 3.

**Table 3.** Implications for Practice, Policy, and Research.

• Neuropsychological impairments in EF – especially in cognitive flexibility, inhibition, and semantic verbal fluency – are prevalent among women victims of IPV and may affect daily autonomy and engagement with interventions.
• Clinicians should consider incorporating routine EF assessments using standardized tools to inform individualized intervention and rehabilitation strategies; identifying EF deficits early may support women’s ability to navigate services and reduce revictimization risk.
• Findings support the need for policy frameworks that recognize cognitive impacts of IPV as part of victims’ support needs, particularly in legal settings; accommodations in testimony procedures may also be relevant.
• Investment in accessible, interdisciplinary care – including neuropsychological services – should be integrated into IPV response programs.
• There is a critical need for longitudinal studies to distinguish pre-existing vulnerabilities from IPV-related cognitive consequences.
• Future research should explore the interplay of IPV, PTSD, mild TBI, and ACEs as co-occurring mechanisms affecting EF; C-PTSD should also be examined as a potential contributing factor.
• Methodological consistency, especially in IPV and EF operationalization, as well as subgroup analyses, are needed to strengthen the evidence base.
• Inclusion of underrepresented groups, such as men and gender-diverse populations, remains a pressing gap in the literature.

*Note.* EF = Executive function; IPV = Intimate partner violence; PTSD = Posttraumatic stress disorder; TBI = Traumatic brain injury; ACEs = Adverse childhood experiences.

## Conclusion

This systematic review and meta-analysis pointed out that women exposed to IPV exhibit lower performance across several EF domains compared with non-victims, especially on cognitive flexibility, inhibition, and semantic verbal fluency. Various potential factors contribute to these alterations: BI, psychopathology, or ACEs. However, the specific contribution of each factor to distinct EF profiles remains underexplored and warrants further investigation, which is essential for tailored interventions and policies.

## Supplemental Material

sj-docx-1-tva-10.1177_15248380251381820 – Supplemental material for Executive Function Correlates of Women Victims of Intimate Partner Violence: A Systematic Review and Meta-Analysis Supplemental material, sj-docx-1-tva-10.1177_15248380251381820 for Executive Function Correlates of Women Victims of Intimate Partner Violence: A Systematic Review and Meta-Analysis by Sara Ferreira-Nascimento, Miguel Rodrigues Barbosa, Magda Sofia Roberto, Sandra Fernandes, Rute Pires and Luís Querido in Trauma, Violence, & Abuse

## Data Availability

Supplemental material for this article is available online. Further information or data can be shared upon request.
