# Supplementary material for: Executive Function Correlates of Women Victims of Intimate Partner Violence: A Systematic Review and Meta-Analysis
Source: Trauma Violence Abuse. 2025 Nov 12;27(5):2336–53. doi: 10.1177/15248380251381820 (PMC13736273; doi:10.1177/15248380251381820)
Supplement: Supplementary file 1 [file sj-docx-1-tva-10.1177_15248380251381820.docx]

**Supplemental Material**

**Table S1**

*PRISMA 2020 Checklist*

| **Section and Topic** | **Item #** | **Checklist item** | **Location where item is reported** |
| --- | --- | --- | --- |
| **TITLE** | | |  |
| Title | 1 | Identify the report as a systematic review. | Title page |
| **ABSTRACT** | | |  |
| Abstract | 2 | See the PRISMA 2020 for Abstracts checklist. | Page 1 |
| **INTRODUCTION** | | |  |
| Rationale | 3 | Describe the rationale for the review in the context of existing knowledge. | Pages 3-4 |
| Objectives | 4 | Provide an explicit statement of the objective(s) or question(s) the review addresses. | Page 4 |
| **METHODS** | | |  |
| Eligibility criteria | 5 | Specify the inclusion and exclusion criteria for the review and how studies were grouped for the syntheses. | Page 5 |
| Information sources | 6 | Specify all databases, registers, websites, organisations, reference lists and other sources searched or consulted to identify studies. Specify the date when each source was last searched or consulted. | Page 5 |
| Search strategy | 7 | Present the full search strategies for all databases, registers and websites, including any filters and limits used. | Page 5-6 and Supplemental Material |
| Selection process | 8 | Specify the methods used to decide whether a study met the inclusion criteria of the review, including how many reviewers screened each record and each report retrieved, whether they worked independently, and if applicable, details of automation tools used in the process. | Pages 6 |
| Data collection process | 9 | Specify the methods used to collect data from reports, including how many reviewers collected data from each report, whether they worked independently, any processes for obtaining or confirming data from study investigators, and if applicable, details of automation tools used in the process. | Pages 6-7 |
| Data items | 10a | List and define all outcomes for which data were sought. Specify whether all results that were compatible with each outcome domain in each study were sought (e.g. for all measures, time points, analyses), and if not, the methods used to decide which results to collect. | Pages 6-7 |
|  | 10b | List and define all other variables for which data were sought (e.g. participant and intervention characteristics, funding sources). Describe any assumptions made about any missing or unclear information. | Pages 6-7 |
| Study risk of bias assessment | 11 | Specify the methods used to assess risk of bias in the included studies, including details of the tool(s) used, how many reviewers assessed each study and whether they worked independently, and if applicable, details of automation tools used in the process. | Pages 7-8 |
| Effect measures | 12 | Specify for each outcome the effect measure(s) (e.g. risk ratio, mean difference) used in the synthesis or presentation of results. | Page 8 |
| Synthesis methods | 13a | Describe the processes used to decide which studies were eligible for each synthesis (e.g. tabulating the study intervention characteristics and comparing against the planned groups for each synthesis (item #5)). | Page 8 and Supplemental Material |
|  | 13b | Describe any methods required to prepare the data for presentation or synthesis, such as handling of missing summary statistics, or data conversions. | Page 8 |
|  | 13c | Describe any methods used to tabulate or visually display results of individual studies and syntheses. | Page 8 |
|  | 13d | Describe any methods used to synthesize results and provide a rationale for the choice(s). If meta-analysis was performed, describe the model(s), method(s) to identify the presence and extent of statistical heterogeneity, and software package(s) used. | Page 8 |
|  | 13e | Describe any methods used to explore possible causes of heterogeneity among study results (e.g. subgroup analysis, meta-regression). | Page 8 |
|  | 13f | Describe any sensitivity analyses conducted to assess robustness of the synthesized results. | Page 8 |
| Reporting bias assessment | 14 | Describe any methods used to assess risk of bias due to missing results in a synthesis (arising from reporting biases). | NA |
| Certainty assessment | 15 | Describe any methods used to assess certainty (or confidence) in the body of evidence for an outcome. | Page 8 |
| **RESULTS** | | |  |
| Study selection | 16a | Describe the results of the search and selection process, from the number of records identified in the search to the number of studies included in the review, ideally using a flow diagram. | Page 8 |
|  | 16b | Cite studies that might appear to meet the inclusion criteria, but which were excluded, and explain why they were excluded. | Page 8 |
| Study characteristics | 17 | Cite each included study and present its characteristics. | Pages 8-18 and Supplemental Material |
| Risk of bias in studies | 18 | Present assessments of risk of bias for each included study. | Pages 18-19 |
| Results of individual studies | 19 | For all outcomes, present, for each study: (a) summary statistics for each group (where appropriate) and (b) an effect estimate and its precision (e.g. confidence/credible interval), ideally using structured tables or plots. | Pages 12-14 and Supplemental Material |
| Results of syntheses | 20a | For each synthesis, briefly summarise the characteristics and risk of bias among contributing studies. | Pages 18-19 |
|  | 20b | Present results of all statistical syntheses conducted. If meta-analysis was done, present for each the summary estimate and its precision (e.g. confidence/credible interval) and measures of statistical heterogeneity. If comparing groups, describe the direction of the effect. | Pages 12-14 |
|  | 20c | Present results of all investigations of possible causes of heterogeneity among study results. | Pages 13-14 |
|  | 20d | Present results of all sensitivity analyses conducted to assess the robustness of the synthesized results. | Pages 13-14 |
| Reporting biases | 21 | Present assessments of risk of bias due to missing results (arising from reporting biases) for each synthesis assessed. | NA |
| Certainty of evidence | 22 | Present assessments of certainty (or confidence) in the body of evidence for each outcome assessed. | Page 14 |
| **DISCUSSION** | | |  |
| Discussion | 23a | Provide a general interpretation of the results in the context of other evidence. | Pages 19-23 |
|  | 23b | Discuss any limitations of the evidence included in the review. | Pages 23-28 |
|  | 23c | Discuss any limitations of the review processes used. | Pages 23-28 |
|  | 23d | Discuss implications of the results for practice, policy, and future research. | Pages 23-28 |
| **OTHER INFORMATION** | | |  |
| Registration and protocol | 24a | Provide registration information for the review, including register name and registration number, or state that the review was not registered. | Page 5 |
|  | 24b | Indicate where the review protocol can be accessed, or state that a protocol was not prepared. | Page 5 |
|  | 24c | Describe and explain any amendments to information provided at registration or in the protocol. | NA |
| Support | 25 | Describe sources of financial or non-financial support for the review, and the role of the funders or sponsors in the review. | Title page |
| Competing interests | 26 | Declare any competing interests of review authors. | Title page |
| Availability of data, code and other materials | 27 | Report which of the following are publicly available and where they can be found: template data collection forms; data extracted from included studies; data used for all analyses; analytic code; any other materials used in the review. | NA |

*Note.* From: Page, M. J., McKenzie, J. E., Bossuyt, P. M., Boutron, I., Hoffmann, T. C., Mulrow, C. D., Shamseer, L., Tetzlaff, J. M., Akl, E. A., Brennan, S. E., Chou, R., Glanville, J., Grimshaw, J. M., Hróbjartsson, A., Lalu, M. M., Li, T., Loder, E. W., Mayo-Wilson, E., McDonald, S., … Moher, D. (2021). The PRISMA 2020 statement: An updated guideline for reporting systematic reviews. *BMJ, 372*(n71), 1–9. https://doi.org/10.1136/bmj.n7

**Table S2**

*Search Equations for Each Database*

| Database | Search equation |
| --- | --- |
| PubMed (NHL) | (female*[Title/Abstract] OR woman[Title/Abstract] OR women[Title/Abstract]) AND ("intimate partner violence"[Title/Abstract] OR "intimate partner abuse"[Title/Abstract] OR "domestic violence"[Title/Abstract] OR "dating violence"[Title/Abstract]) AND ("executive dysfunction*"[Title/Abstract] OR "executive function*"[Title/Abstract] OR "executive impairment*"[Title/Abstract] OR "executive deficit*"[Title/Abstract] OR "executive control"[Title/Abstract] OR neuropsycholog*[Title/Abstract] OR neurocogniti*[Title/Abstract] OR cogniti*[Title/Abstract] OR neurolog*[Title/Abstract] OR neural[Title/Abstract]) NOT (review[Title/Abstract] OR meta-analysis[Title/Abstract] OR "case study"[Title/Abstract] OR "case report"[Title/Abstract] OR "case series"[Title/Abstract]) |
| Web of Science Core Collection (Clarivate) | (female* OR woman OR women) AND (“intimate partner violence” OR "intimate partner abuse” OR "domestic violence” OR "dating violence”) AND (“executive dysfunction*” OR "executive function*” OR "executive impairment*” OR "executive deficit*” OR "executive control” OR neuropsycholog* OR neurocogniti* OR cogniti* OR neurolog* OR neural) NOT (review OR meta-analysis OR “case study” OR “case report” OR “case series”) |
| PsycINFO (EBSCO) |  |
| Scopus (Elsevier) | TITLE-ABS((female* OR woman OR women) AND ("intimate partner violence" OR "intimate partner abuse" OR "domestic violence" OR "dating violence") AND ("executive dysfunction*" OR "executive function*" OR "executive impairment*" OR "executive deficit*" OR "executive control" OR neuropsycholog* OR neurocogniti* OR cogniti* OR neurolog* OR neural) AND NOT (review OR meta-analysis OR "case study" OR "case report" OR "case series")) |

**Table S3**

*Characteristics of Included Studies*

| Author(s) and year; study country and design | Participants by group of women (sample size and age in years) | Identification of IPV | Prevalence of BI among IPV victims | Prevalence of MH outcomes and ACEs among IPV victims | Executive functions assessed | Neuropsychological tests used | Results |
| --- | --- | --- | --- | --- | --- | --- | --- |
| Aupperle et al., 2012; United States of America (USA); Cross-sectional | **Victims IPV-related PTSD+:**  *n* = 37*; M* = 38.32 ± 9.13 **Non-victims PTSD–:**  *n* = 33*; M* = 37.76 ± 11.13 | **Past physical and/or sexual abuse** within the last 5 years but having ended at least one month before enrollment in the study; general community, hospitals, psychiatric clinics | 0% loss of consciousness longer than 10 minutes (exclusion criterion) | **PTSD:** 100% current full or partial diagnosis (inclusion criterion) **Depression:** Not reported **Anxiety and ACEs:** Not assessed | **Inhibition**  **Cognitive flexibility** (attentional switching) **Reasoning** (abstract reasoning) | Delis-Kaplan Executive Function System (D-KEFS) - Color-Word Interference Inhibition  D-KEFS - Color-Word Inhibition/Switching  Wisconsin Card Sorting Test (WCST) | **↓ Performance for women IPV-related PTSD+ vs. non-victims** in cognitive flexibility (Color-Word Inhibition/Switching) (*p* = .03) |
| Aupperle et al., 2016; USA; Cross-sectional | **Victims IPV-related PTSD+:**  *n* = 10*; M* = 34.60 ± 9.40 **Non-victims PTSD–:**  *n* = 12*; M* = 35.25 ± 6.44 | **Experience of IPV** within the last 5 years; general community and IPV-related services; self-reported by phone interview | 0% loss of consciousness longer than 10 minutes (exclusion criterion) | **PTSD:** 100% current full or partial diagnosis (inclusion criterion) **Depression and anxiety:** Not assessed **ACEs:** Not assessed; 40% of women PTSD+ | **Cognitive flexibility** (attentional switching)  **Inhibition** | D-KEFS - Color-Word Inhibition/Switching D-KEFS - Color-Word Interference Inhibition | No sig. differences for EF tests between groups No sig. correlations between PTSD severity and EF tests |
| Chung et al., 2014; Taiwan; Cross-sectional | **IPV victims:**  *n* = 45 **- PTSD+:**  *n* = 28*; M* = 36.68 ± 9.14 **- PTSD–:**  *n* = 17*; M* = 42.00 ± 12.12 **Non-victims PTSD–:**  *n* = 30*; M* = 37.17 ± 11.56 | **Past IPV**, having ended at least 1 month before enrollment in the study; center for IPV prevention | 0% serious brain injury (exclusion criterion) | **PTSD:** 62% above current severity threshold **Depression:** Not reported **Anxiety:** Not reported **ACEs:** Not assessed | **Cognitive flexibility** (mental flexibility)  **Semantic verbal fluency** (category fluency)  **Working memory** | Modified Card Sorting Test (MCST) Category Fluency (fruits, four-legged animals, and vegetables)  Wechsler Adult Inteligence Scale - 3rd edition (WAIS-III) - Digit Span (total score, forward or backward not specified)  Rey Auditory Verbal Learning Test (RAVLT) | **↓ Performance for IPV victims PTSD+ vs. victims PTSD–** in cognitive flexibility (MCST) (*p* < .01)  - PTSD severity positively correlated with cognitive flexibility (MCST) (*p* < .01) and negatively correlated with semantic verbal fluency (Category Fluency) (*p* < .05) |
| Dabkowska, 2007; Poland; Cross-sectional | **IPV victims:** *N* = 63*; M* = 35.65 ± 10.4; Age range: 17-55 | **Past physical and mental/emotional abuse**; IPV-related hostel and/or other services | Not assessed | **PTSD:** 97.8% symptoms of varying intensity levels; 48.9% extreme or severe levels **Depression, anxiety, and ACEs:** Not assessed | **Cognitive flexibility** (set-shifting) | Trail Making Test (TMT) Part B (TMT-B) | **IPV victims** (age ≥ 35 years old): - TMT-B: *M* = 104.8; *SD* = 75.7 (time in seconds) |
| Daugherty et al., 2018; Spain; Cross-sectional | **IPV victims:**  **- Psychological abuse (PA):**  *n* = 24*; M* = 41.08 ± 6.77; Age range: 24-52 **- Physical and psychological abuse (PPA):**  *n* = 45*; M* = 39.36 ± 8.43; Age range: 18-52  **Non-victims:** *n* = 39*; M* = 39.49 ± 8.67; Age range: 20-52 | **Past or current psychological and/or physical abuse**; nongovernmental organizations; semistructured interview; Revised Conflict Tactic Scale (CTS2) | 0% TBI (exclusion criterion) | **PTSD:** 0% (exclusion criterion) **Depression:** 0% (exclusion criterion) **Anxiety and ACEs:** Not assessed | **Working memory** (updating/monitoring) **Inhibition**  **Cognitive flexibility**  **Decision-making  Planning**  **Reasoning** (abstract reasoning) | WAIS-III - Letters & Numbers  Five Digit Test (FDT) - Part 3 FDT - Part 4 D-KEFS - TMT - Part 4 Iowa Gambling Task (IGT) Behavioral Assessment of the Dysexecutive Syndrome (BADS) - Map of the Zoo  Kaufman Brief Intelligence Test (K-BIT) - Matrix  Reasoning | **↓ Performance for** **PPA vs. non-victims** in inhibition (FDT-3) (*p* = .02), planning (Map of the Zoo_Time) (*p* = .02) and reasoning (Matrix Reasoning) (*p* = .03)  **↓ Performance for** **PA and PPA vs. non-victims** in decision-making (IGT) (*p* = .01) **↓ Performance for PPA vs. PA** in cognitive flexibility (FDT-4) (*p* = .03) |
| Fennema-Notestine et al., 2002; USA; Cross-sectional | **IPV victims:** *n* = 22*; M* = 34.4 ± 9.8; Age range: 19-49 **- PTSD+:** *n* = 11*; M* = 33.5 ± 10.3; Age range: 19-49 **- PTSD–:** *n* = 11*; M* = 35.4 ± 9.6; Age range: 22-48 **Non-victims PTSD–:** *n* = 17*; M* = 35.3 ± 12.5; Age range: 19-57 | **Physical, psychological and/or sexual abuse**; services specializing in IPV; CTS2 | 0% serious head injury (resulting in hospitalization) | **PTSD:** 28% current diagnosis **Depression:** Not reported **Anxiety:** Not reported **ACEs:** Not reported | **Cognitive flexibility** (referred to only as EF) **Working memory** (auditory working memory) | TMT-B  Paced Auditory Serial Addition Task (PASAT) | **IPV victims overall:** - Cognitive flexibility (TMT-B) negatively correlated with cortical gray matter (*p* = .05)  - Working memory (PASAT) positively correlated with mesial temporal gray matter volume (*p* < .05)  - Supratentorial cranial vault volume negatively correlated with ACEs - Physical Abuse (*p* < .05) |
| Fillol et al., 2023; Spain; Cross-sectional | **IPV victims:** *n* = 37*; M* = 36.19 ± 9.03; Age range: 21-60 **Non-victims:** *n* = 23*; M* = 31.43 ± 11.84; Age range: 18-60 | **Physical, psychological or sexual violence**; general community; Composite Abuse Scale Revised - Short Form (CASR-SF) | Not assessed | **PTSD:** Not assessed **Depression, anxiety and ACEs:** Assessed, but not reported | **Working memory** | WAIS-III - Digit Span (forward and backward) | **↓ Performance for IPV victims vs. non-victims** in working memory - Digit Span forward score (*p* = .009) and Digit Span forward longest correct series (*p* = .004) |
| Ipiña & Cortés, 2023; Mexico; Cross-sectional | **IPV victims:** *N* = 14*; M* = 34.86 ± 9.18; Age range: 21-54 | **Past physical, psychological or sexual abuse in the last year**; governmental institutions for IPV victims | Not assessed | **PTSD:** 50% partial diagnosis **Depression:** 42.86% moderate **Anxiety:** 71.43% moderate to severe  **ACEs:** Not assessed | **Executive functions** (domains not specified) | Brief Neuropsychological Evaluation in Spanish - NEUROPSI | **↓ Performance for IPV victims PTSD+ vs. victims PTSD–** in EF tests (*p* = .007) - PTSD severity correlated negatively with EF performance |
| Lee & DePrince, 2017; USA; Cohort | **IPV victims:**  *N* = 236*; M* = 33.4 ± 11.0; Age range: 18-63 | **Police-reported physical assault**; community coordinated response programs; CTS2 | Not assessed | **PTSD:** Not assessed **Depression, anxiety, and ACEs:** Not assessed | **Inhibition** (interference control; selective attention)  **Working memory** | Stroop Color-Naming Task  Wechsler Adult Intelligence Scale - 4th edition (WAIS-IV) - Letter-Number Sequencing | **IPV victims:** - Working memory (Letter-Number Sequencing): *M* = 9.14, *SD* = 3.04 - Inhibition (Stroop Task): *M* = 31.85 seconds, *SD* = 11.38 |
| Maldonado-Rodriguez et al., 2021; Canada; Cross-sectional | **IPV victims:** *N* = 40*; M* = 37.4 ± 7.97 | **Experience of IPV**; local shelters and organizations; Women’s Experiences with Battering (WEB) | 95% IPV-related; 75% non-related | **PTSD:** 95% elevated levels of symptoms **Depression:** 62% elevated levels  **Anxiety:** 58% elevated levels  **ACEs:** Not assessed | **Cognitive flexibility** | TMT-B Object Hit and Avoid (OHA) Task for sensorimotor function | **Victims with IPV-related BI:**  - Negative correlations between sensorimotor function (OHA) and cognitive flexibility (TMT-B) (*p* = .008). - Sensorimotor function (OHA) associated with PTSD and depression |
| Meneses et al., 2023; Ecuador; Cross-sectional | **IPV victims:** *N* = 120*; M* = 34.3 ± 0.7 **- Psychological violence:** *n* = 56*; M* = 35.4 ± 1.1 **- Physical violence:** *n* = 52*; M* = 33.6 ± 1.1 **- Sexual violence:** *n* = 12; *M* = 32.3 ± 2.9 | **Physical, psychological or sexual violence**; consultations and outpatient care of a psychology service of a Health Center; Technical Standard for Comprehensive Attention to Gender Violence | 0% acquired brain damage (exclusion criteria) | **PTSD:** 40.8% current probable diagnosis **Depression:** 26.7% clinical level **Anxiety:** 60% clinical level **ACEs:** Not assessed | **Working memory**  **Cognitive** **flexibility**  **Inhibition** (complex and selective attention) **Phonological verbal fluency**  **Semantic verbal fluency** | WAIS-IV - Digits TMT-B Stroop Test  Phonological verbal fluency test (P-M-R) Semantic verbal fluency test | **IPV victims:**  - Clinically sig. values in working memory (Digits) (*Z* = −1.67)  - Lower values than the average for their age and cultural level in cognitive flexibility (TMT-B) (*Z* = −1.15), inhibition (Stroop Test) (*Z* = −0.20), and semantic verbal fluency test (*Z* = −1.23) **Sexual violence group:**  - Clinically sig. values (*Z* < −1,5) in cognitive flexibility (TMT-B) (*Z* = −2.40) |
| Molinares et al., 2023; Colombia; Cross-sectional | **IPV victims:**  *N* = 70*; M* = 29.6 ± 11.4 | **Physical, sexual or psychological abuse**; nongovernmental organizations, including social services centers and shelters, and general community; semistructured interview | 31.4% IPV-related; 2.9% non-related | **PTSD:** 37% above clinical cutoff **Depression:** 71% clinical level **Anxiety:** 80% clinical level **ACEs:** Not assessed | **Working memory  Cognitive flexibility** | WAIS-IV - Digit Span (backward) Army Individual Test Battery - TMT-B | **- BI severity** negatively correlated with working memory (Digit Span) (*p* = .022) and positively with cognitive flexibility (TMT-B) (*p* = .036)  - PTSD severity positively correlated with cognitive flexibility (TMT-B) (*p* = .044) - BI severity remained sig. associated with working memory (Digit Span) (*p* = .026) after controlling for PTSD severity; the association with cognitive flexibility (TMT-B) lost sig. |
| Muir et al., 2022; USA; Cohort | **Victims of IPV-related strangulation** *N* = 8*; M* = 27.00 ± 12.80 | **Strangulation in the last 7 days**; hospital, general community, and shelters; sociodemographic questionnaire | 100% IPV-related strangulation | **PTSD:** Not assessed **Depression:** Not reported **Anxiety:** Not reported **ACEs:** Not assessed | **Inhibition** (attention; inhibitory control)  **Cognitive flexibility** (speeded mental flexibility) | Flanker Inhibitory Control and Attention Test  TMT-B | **Victims of IPV-related strangulation**: - Inhibition (Flanker): *M* = 73.4, *SD* = 32.96 - Cognitive flexibility (TMT-B): *M* = 8.5, *SD* = 1.68 |
| Raskin et al., 2023; USA; Cross-sectional | **IPV victims:**  *n* = 50; Age range: 18-65  **Non-victims:** *n* = 50; Age range: 18-65 **Sexual assault victims (non-IPV):** *n* = 35; Age not reported | **Physical, sexual, or emotional abuse**; shelters, support groups, and hospitals; self-reported by questionnaire | 95% possible BI; 60% possible TBI | **PTSD:** 43% current diagnosis **Depression:** 23% severe **Anxiety:** 24% severe **ACEs:** Not assessed | **Semantic verbal fluency** (verbal fluency)  **Global verbal fluency** (verbal fluency)  **Cognitive flexibility** (referred to only as EF)  **Inhibition** (referred to only as EF) | Boston Diagnostic Aphasia Examination (BDAE) - Animal Naming  Controlled Oral Word Association Test (COWAT)  TMT-B  Stroop Color-Word Interference Test | **↓ Performance for IPV victims vs. non-victims** in semantic verbal fluency (Animal Naming) (*p* < .01), cognitive flexibility (TMT-B) (*p* < .05), inhibition (Stroop Test) (*p* < .05) **↓ Performance for IPV victims who reported non-fatal strangulation vs. who did not** in semantic verbal fluency (Animal Naming) |
| Seedat et al., 2005; USA; Cross-sectional | **IPV victims:** *n* = 16; *M* = 32.3 ± 9.9; Age range: 20.0-49.0 **- PTSD+** *n* = 7*; M* = 30.1 ± 10.9; Age range: 20.0-49.0 **- PTSD–** *n* = 9*; M* = 35.9 ± 9.1; Age range: 21.0-48.0 **Non-victims PTSD–:** *n* = 11*; M* = 34.9 ± 9.3; Age range: 19.0-44.0 | **Past psychological, physical or sexual abuse**, having ended for at least 4 months but not longer than 2 years; services for IPV victims; CTS2 | 0% head injury associated with cognitive dysfunction (exclusion criterion) | **PTSD:** 25.9% current diagnosis **Depression and anxiety:** Not reported **ACEs:** Not assessed | **Cognitive flexibility** (set-shifting)  **Inhibition** (cognitive interference) | TMT-B  Stroop Color-Word Interference Test | **↓ Performance for IPV victims vs. non-victims** in inhibition (Stroop Test) (*p* = .001) and cognitive flexibility (TMT-B) (*p* = .012) |
| Stein et al., 2002; USA; Cross-sectional | **IPV victims:**  *n* = 39 **- IPV-related PTSD+:** *n* = 17*; M* = 33.8 ± 9.8 **- PTSD–:** *n* = 22*; M* = 34.5 ± 9.0 **Non-victims PTSD–:** *n* = 22*; M* = 29.4 ± 10.7 | **Past physical and/or sexual abuse**, having ended at least 4 weeks but no longer than 2 years before enrollment in the study; services specializing in IPV; CTS2 | 0% loss of consciousness greater than 10 minutes (exclusion criterion) | **PTSD:** 43% current diagnosis **Depression:** Not reported **Anxiety and ACEs:** Not assessed | **Semantic verbal fluency** (also identified as language)  **Phonological verbal fluency** (language)  **Working memory** (auditory speeded sustained attention)  **Cognitive flexibility** (set-shifting)  **Inhibition**  **Planning** (organization) | Category Test COWAT (Animals, fruits, vegetables)  COWAT (FAS)  WAIS-III - Digit Span (forward, backward and total) Auditory Consonant Trigrams Digit Vigilance Test PASAT TMT-B and TMT B-A  Stroop Color-Word Interference Test  Rey-Osterrieth Complex Figure Test | **↓ Performance for IPV victims PTSD+ and – vs. non-victims** in inhibition (Stroop Test) (*p* = .005) and working memory (PASAT) (*p* = .02) **↓ Performance for IPV victims IPV-related PTSD+ vs. non-victims** in cognitive flexibility (TMT-B and TMT B-A) (*p* = .01 and *p* = .03) **↓ Performance for IPV victims PTSD– vs. non-victims** in semantic verbal fluency (Category Test) (*p* = .03) |
| Twamley et al., 2009; USA; Cross-sectional | **Victims IPV-related PTSD+:**  *n* = 55*; M* = 36.7 ± 8.8 **Non-victims PTSD–:** *n* = 20*; M* = 35.0 ± 8.6 | **Experience of IPV** within the last 5 years; general community and agencies specialized in IPV; self-reported by phone interview | 0% loss of consciousness longer than 10 minutes or requiring hospitalization for at least 24 hours (exclusion criterion) | **PTSD:** 100% current full or partial diagnosis (inclusion criterion) **Depression and anxiety:** Not reported **ACEs:** Not reported | **Inhibition**  **Cognitive flexibility** (switching)  **Reasoning**  **Phonological verbal fluency** (speeded fluency)  **Semantic verbal fluency** (speeded fluency) **Nonverbal fluency** (speeded fluency) **Planning** (visuoconstruction) | D-KEFS - Color-Word Interference Test Inhibition vs. Color Naming D-KEFS - Switching subtests contrasting scores  WCST  D-KEFS - Letter Fluency  D-KEFS - Category Fluency  D-KEFS - Design Fluency  Rey-Osterrieth Complex Figure Test | **↓ Performance for victims IPV-related PTSD+ vs. non-victims** in phonological verbal fluency (Letter Fluency) (*p* = .002), nonverbal fluency (Design Fluency) (*p* = .005) and reasoning (WCST) (*p* = .020)  - More severe ACEs associated with worse performance in cognitive flexibility (Category Fluency Switching vs. Category Fluency) (*p* = .005) |
| Valera & Berenbaum, 2003; USA; Cross-sectional | **IPV victims:** *n* = 99*; M* = 32.0 ± 9.3; Age range: 18-54 **- TBI subgroup** *n* = 57*; M* = 31.2 ± 9.0; Age range: 18-52 | **Past or current physical abuse**; shelters, IPV and substance abuse programs, and general community; Conflict Tactics Scale (CTS); Severity of Violence Against Women Scale (SVAWS) | 74% mild, moderate or severe IPV-related TBI; 27% non-related (full sample) | **PTSD:** Not reported **Depression and anxiety:** Not reported  **ACEs:** Not assessed | **Cognitive flexibility** **Working memory** (attention; short-term memory)  **Nonverbal fluency** (design fluency) | TMT-B  Wechsler adult intelligence scale - Revised (WAIS-R) - Digit Span (forward and backward)  Ruff Figural Fluency Test (RFFT) | **IPV-related BI severity** negatively correlated with cognitive flexibility (TMT-B) (*p* < .05) even when controlling for anxious arousal (*p* < .05); the correlation lost sig. when controlling for PTSD severity |
| Valera & Kucyi, 2016; USA; Cross-sectional | **IPV victims:** *N* = 20*; M* = 33.9 ± 11.6 | **Physical abuse;** shelters, IPV programs, and general community; CTS | BI: 100% at least one IPV-related TBI | **PTSD:** Not reported **Depression and anxiety:** Not reported **ACEs:** Not reported | **Cognitive flexibility** | TMT-B | No sig. correlations between IPV-related BI severity and cognitive flexibility (TMT-B) |
| Valera et al., 2019; USA; Cross-sectional | **IPV victims:** *N* = 20*; M* = 32.3 ± 10.9 | **Physical abuse**; shelters, IPV programs, and general community; CTS; SVAWS | 100% IPV-related mild TBI | **PTSD:** Not reported **Depression and anxiety:** Not reported  **ACEs:** Not reported | **Cognitive flexibility** | TMT-B | No sig. correlations between fractional anisotropy in the white matter (associated with brain injury score) and cognitive flexibility (TMT-B) |
| Valera et al., 2022; USA; Cross-sectional | **IPV victims:** *N* = 99*; M* = 32 ± 9.3; Age range: 18-54 **- Strangulation-related AICs subgroup** *n* = 52*; M* = 31.4 ± 9.2; Age range: 18-52 | **Past or current physical abuse**; shelters, IPV and substance abuse programs, and general community; CTS; SVAWS | 27% (total group) and 29% (subgroup) strangulation-related AICs from IPV | **PTSD:** Not reported **Depression and anxiety:** Not reported  **ACEs:** Not reported | **Cognitive flexibility**  **Working memory** (short-term memory)  **Nonverbal fluency** (nonverbal fluency) | TMT-B WAIS-R - Digit Span (forward and backward) RFFT | **↓ Performance for IPV victims with strangulation-related AIC vs. without** in working memory (Digit Span) when controlling for ACEs, PTSD, IPV-related mild TBIs and depression - Cognitive flexibility (TMT-B) and nonverbal fluency (RFFT) maintained without sig. differences even when controlling for PTSD, depression and IPV-related mild TBIs |
| Williams et al., 2017; USA; Cohort | **IPV victims:** *N* = 11; Age range: 30-63 | **Sexual or physical abuse**; general community; semistructured interview | Not assessed | **Depression:** Not reported **PTSD, anxiety and ACEs:** Not assessed | **Cognitive flexibility** (also identified as processing speed)  **Semantic verbal fluency** | TMT-B  Animal Naming Task | Descriptive statistics not reported |

*Note.* IPV = Intimate partner violence; PTSD = Posttraumatic stress disorder; PTSD+ = With PTSD; PTSD**– =** Without PTSD; *N* = Total sample size; *n* = Subsample size; *M* = Mean; *SD* = Standard deviation; BI = Brain injury; TBI = Traumatic brain injury; AICs = Alterations in consciousness; MH = Mental health; ACEs = Adverse childhood experiences; EF = Executive function.

For each executive function, the name in parentheses corresponds to the term used by the authors in each article.

Only significant results were presented unless any significant results were found.

**Table S4**

*Assessment Methods for Brain Injury and Posttraumatic Stress Disorder*

| Study | Assessment methods for BI | Assessment methods for PTSD |
| --- | --- | --- |
| Aupperle et al., 2012 | History of loss of consciousness longer than 10 minutes as exclusion criterion  Method of assessment not specified; possibly based on self-report | Clinician-Administered PTSD Scale for DSM-IV (CAPS-IV) used to establish a current full or partial PTSD diagnosis related to IPV |
| Aupperle et al., 2016 | History of loss of consciousness longer than 10 minutes as exclusion criterion  Method of assessment not specified; possibly based on self-report | CAPS-IV used to establish a current full or partial PTSD diagnosis related to IPV and assess symptom severity  PTSD Checklist (PCL) used to assess PTSD symptom severity |
| Chung et al., 2014 | History of medical or organic disease in the central nervous system (e.g., epilepsy, a tumor or serious BI) as exclusion criterion  Method of assessment not specified; possibly based on self-report | Chinese version of the Davidson Trauma Scale (DTS-C) used to establish a current PTSD diagnosis and assess symptom severity |
| Dabkowska, 2007 | Not assessed | PTSD Interview (PTSD-I) used to establish a PTSD diagnosis related to IPV and assess symptom severity |
| Daugherty et al., 2018 | Self-reported (clinical interview) history of TBI as exclusion criterion | Millon Clinical Multiaxial Inventory (MCMI-III) used to screen for PTSD as exclusion criterion |
| Fennema-Notestine et al., 2002 | History of serious head injury (resulting in hospitalization) as exclusion criterion  Method of assessment not specified; possibly based on self-report | Structured Clinical Interview for DSM-IV (SCID) and CAPS-IV used to establish a current PTSD diagnosis  CAPS-IV and Impact of Event Scale-Revised (IES-R) used to assess PTSD symptom severity |
| Fillol et al., 2023 | Not assessed | Not assessed |
| Ipiña & Cortés, 2023 | Not assessed | PTSD checklist for DSM-V (PCL-5) used to establish a full or partial PTSD diagnosis and assess symptom severity |
| Lee & DePrince, 2017 | Not assessed | Not assessed |
| Maldonado-Rodriguez et al., 2021 | Brain Injury Severity Assessment (BISA) used to assess presence, recency, frequency, and severity of IPV-related and non-related BI | CAPS-IV used to assess PTSD symptom severity |
| Meneses et al., 2023 | History of acquired brain damage as exclusion criterion  Method of assessment not specified; possibly based on self-report | PCL-5 used to establish a current PTSD diagnosis and assess symptom severity |
| Molinares et al., 2023 | BISA used to assess presence, recency, frequency, and severity of IPV-related and non-related BI | PCL-5 used to establish a PTSD diagnosis and assess symptom severity |
| Muir et al., 2022 | Revised Head Injury Scale (HIS-r) used to assess quantity, duration, and severity of concussion-related symptoms  History of IPV-related non-fatal strangulation assessed by self-report among women referred from the emergency department of a trauma center within an academic medical hospital | Not assessed |
| Raskin et al., 2023 | HELPS Screening Tool and IPV Health History Questionnaire (IHHQ) used to determine possible history of BI, including TBI  IHHQ used to assess post-concussion symptoms  History of non-fatal strangulation assessed by self-report | Clinician-Administered PTSD Scale for DSM-5 (CAPS-5) used to establish a current PTSD diagnosis and assess symptom severity |
| Seedat et al., 2005 | History of head injury associated with cognitive dysfunction as exclusion criterion  Method of assessment not specified; possibly based on self-report | SCID used to establish a current PTSD diagnosis |
| Stein et al., 2002 | History of head injury (with loss of consciousness greater than 10 min) as exclusion criterion  Method of assessment not specified; possibly based on self-report | CAPS-IV used to establish a current PTSD diagnosis related to IPV and assess symptom severity  PTSD module of the Structured Clinical Interview for the DSM-IV (SCID-P) to establish a lifetime PTSD diagnosis for any trauma other than IPV  IES-R to assess current PTSD symptom severity |
| Twamley et al., 2009 | Self-reported (phone interview) history of loss of consciousness longer than 10 minutes or requiring hospitalization for at least 24 hours as exclusion criterion | PTSD Checklist - Civilian Version (PCL-C) to quantify PTSD symptoms and assess the likelihood of PTSD  CAPS-IV to establish a current full or partial PTSD diagnosis and assess symptom severity |
| Valera & Berenbaum, 2003 | Semistructured interview used to assess IPV-related and non-related TBI and strangulation-related AICs, based on the diagnostic criteria by the American Congress of Rehabilitation Medicine Special Interest Group on Mild TBI  Interview referred to as BISA in a subsequent article | Clinician-Administered PTSD Scale for DSM–IV – One-Week Symptom Status Version (CAPS-2) to assess current PTSD symptom severity |
| Valera & Kucyi, 2016 | Semistructured interview used to assess IPV-related and non-related TBI and strangulation-related AICs, based on the diagnostic criteria by the American Congress of Rehabilitation Medicine Special Interest Group on Mild TBI  Interview referred to as BISA in a subsequent article | CAPS-2 to assess current PTSD symptom severity |
| Valera et al., 2019 | Semistructured interview used to assess IPV-related and non-related TBI and strangulation-related AICs, based on the diagnostic criteria by the American Congress of Rehabilitation Medicine Special Interest Group on Mild TBI  Interview referred to as BISA in a subsequent article | CAPS-2 to assess current PTSD symptom severity |
| Valera et al., 2022 | BISA used to assess IPV and non-related TBI and strangulation-related AICs | CAPS-2 to assess current PTSD symptom severity |
| Williams et al., 2017 | Not assessed | Not assessed |

*Note*. BI = Brain injury; PTSD = Posttraumatic stress disorder; TBI = Traumatic brain injury; IPV = Intimate partner violence; AICs = Alterations in consciousness.

**Table S5**

*Neuropsychological Tests Used in Included Studies, by Executive Function Domain and Frequency*

| Executive function domain | Neuropsychological tests used and frequency |
| --- | --- |
| Cognitive flexibility | Trail Making Test - Part B (TMT-B); 14 studies |
|  | Delis-Kaplan Executive Function System (D-KEFS) - Color-Word Inhibition/Switching; 2 studies |
|  | Trail Making Test - Part B-A; 1 study |
|  | Modified Card Sorting Test (MCST); 1 study |
|  | Five Digit Test (FDT) - Part 4; 1 study  D-KEFS - TMT - Part 4; 1 study |
|  | D-KEFS - Switching subtests contrasting scores; 1 study |
| Inhibition | Stroop Color and Word Test; 5 studies |
|  | D-KEFS - Color-Word Interference Inhibition; 2 studies |
|  | FDT - Part 3; 1 study |
|  | Flanker Inhibitory Control and Attention Test; 1 study |
| Working memory | Wechsler Adult Intelligence Scale (WAIS) - Digit Span Subtest; 6 studies |
|  | Paced Auditory Serial Addition Task (PASAT); 2 studies |
|  | WAIS-III - Letters & Numbers; 1 study |
|  | Rey Auditory Verbal Learning Test (RAVLT) - First Trial; 1 study |
|  | Auditory Consonant Trigrams; 1 study |
|  | Digit Vigilance Test; 1 study |
| Semantic verbal fluency | Category Fluency; 2 studies |
|  | Boston Diagnostic Aphasia Examination (BDAE) - Animal Naming; 1 study |
|  | Semantic Verbal Fluency Test; 1 study |
|  | Controlled Oral Word Association Test (COWAT) – Category Test; 1 study |
| Phonological verbal fluency | Phonological Verbal Fluency Test; 1 study |
|  | D-KEFS - Letter Fluency; 1 study |
|  | COWAT - FAS; 1 study |
| Global verbal fluency | COWAT; 1 study |
| Nonverbal fluency | Ruff Figural Fluency Test (RFFT); 2 studies |
|  | D-KEFS - Design Fluency; 1 study |
| Planning | Rey–Osterrieth Complex Figure Test; 2 studies |
|  | Behavioral Assessment of the Disexecutive Syndrome (BADS) - Map of the Zoo; 1 study |
| Reasoning | Wisconsin Card Sorting Test (WCST); 2 studies |
|  | Kaufman Brief Intelligence Test (K-BIT) - Matrix Reasoning; 1 study |
| Decision-making | Iowa Gambling Task (IGT); 1 study |

*Note*. This table includes data from all studies, whether comparing IPV victims with non-victims or examining executive function performance in relation to other variables (e.g., PTSD, BI severity) within the IPV group.

**Table S6**

*Original and Reclassified Executive Function or Cognitive Domains of Neuropsychological Tests*

| Study | Neuropsychological test | Original EF/cognitive domain(s) | EF/cognitive domain(s) reclassification | Justification |
| --- | --- | --- | --- | --- |
| Aupperle et al., 2012 | Wisconsin Card Sorting Test (WCST) | Abstract reasoning and cognitive flexibility | Reasoning | Classified as a measure of both abstract reasoning and cognitive flexibility in one study and exclusively as a measure of reasoning in another study |
| Dabkowska, 2007 | Trail Making Test - Part B (TMT-B) | Cognitive flexibility and working memory | Cognitive flexibility | Classified as a measure of both cognitive flexibility and working memory in one study and exclusively as a measure of cognitive flexibility (or set-shifting/mental flexibility) in 11 studies |
| Maldonado Rodriguez et al., 2021 | Trail Making Test - Part A (TMT-A) | Classified as a measure of EF without specifying the domain | Results not included in the review | Classified as an EF in one study and as a measure of visuomotor processing speed in eight studies |
| Stein et al., 2002 | Controlled Oral Word Association Test (COWAT) | Language | Verbal fluency | Respective semantic and phonological verbal fluency subtests included in one study and identified as verbal fluency in another study |

*Note*. EF = Executive function.

**Table S7**

*Characteristics of the Studies Included in the Meta-Analysis*

| Study by domain | Test | IPV victims | | | Non-victims | | | PTSD diagnosis | Moderate/severe BI history |
| --- | --- | --- | --- | --- | --- | --- | --- | --- | --- |
|  |  | *M* | *SD* | *n* | *M* | *SD* | *n* |  |  |
| Cognitive flexibility |  |  |  |  |  |  |  |  |  |
| Raskin et al., 2023 | TMT-B (completion time) | 70.82 | 22.58 | 50 | 55.96 | 19.92 | 50 | + / – | + / – |
| Seedat et al., 2005 | TMT-B (completion time) | 77.2 | 35.2 | 16 | 46.4 | 14.5 | 11 | + / – | – |
| Stein et al., 2002 | TMT-B (completion time) | 95.1 | 61.9 | 17 | 55.0 | 18.3 | 22 | + | – |
| Inhibition |  |  |  |  |  |  |  |  |  |
| Raskin et al., 2023 | Stroop Test (Interference accuracy) | 42.02 | 11.55 | 50 | 47.87 | 9.71 | 50 | + / – | + / – |
| Seedat et al., 2005 | Stroop Test (Interference accuracy) | 41.7 | 8.3 | 16 | 54.3 | 0 | 11 | + / – | – |
| Stein et al., 2002 | Stroop Test (Interference accuracy) | 41.8 | 8.6 | 17 | 50.9 | 9.3 | 22 | + | – |
| Working memory |  |  |  |  |  |  |  |  |  |
| Chung et al., 2014 | WAIS-III - Digit Span (accuracy) | 21.36 | 4.64 | 28 | 21.73 | 3.66 | 30 | + | – |
| Fillol et al., 2023 | WAIS-III - Digit Span (Forward accuracy) | 7.65 | 1.65 | 37 | 8.78 | 1.44 | 23 | Not assessed | Not assessed |
| Stein et al., 2002 | WAIS-III - Digit Span (Total accuracy) | 15.2 | 4.1 | 17 | 16.5 | 3.3 | 22 | + | – |
| Semantic verbal fluency |  |  |  |  |  |  |  |  |  |
| Chung et al., 2014 | Category Fluency (accuracy) | 40.14 | 9.14 | 28 | 43.23 | 8.89 | 30 | + | – |
| Raskin et al., 2023 | BDAE - Animal Naming (accuracy) | 20.85 | 6.98 | 50 | 25.03 | 5.69 | 50 | + / – | + / – |
| Twamley et al., 2009 | D-KEFS - Category Fluency (accuracy) | 11.0 | 3.3 | 55 | 11.8 | 3.5 | 20 | + | – |

*Note.* IPV = Intimate partner violence; PTSD = Posttraumatic stress disorder; BI = Brain injury; *M* = Mean; *SD* = Standard deviation; *n* = Sample size; TMT-B = Trail Making Test – Part B; WAIS-III = Wechsler Adult Intelligence Scale – 3^rd^ Edition; BDAE = Boston Diagnostic Aphasia Examination; D-KEFS = Delis-Kaplan Executive Function System.

+ With PTSD diagnosis or moderate/severe BI history; – Without PTSD diagnosis or moderate/severe BI history.

**Table S8**

*Characteristics of the Additional Studies Included in the Sensitivity Analysis*

| Study by domain | Test | IPV victims | | | Non-victims | | | PTSD diagnosis | Moderate/severe BI history |
| --- | --- | --- | --- | --- | --- | --- | --- | --- | --- |
|  |  | *M* | *SD* | *n* | *M* | *SD* | *n* |  |  |
| Cognitive flexibility |  |  |  |  |  |  |  |  |  |
| Aupperle et al., 2012 | D-KEFS - Color-Word Inhibition/Switching scaled score*^1^ (acccuracy) | 9.59 | 3.17 | 37 | 11.39 | 2.30 | 33 | + | – |
| Chung et al., 2014 | MCST (number of errors) | 4.89 | 5.88 | 28 | 3.39 | 4.65 | 30 | + | – |
| Daugherty et al., 2018 | D-KEFS - TMT-4 (completion time) | 122.69 | 56.34 | 45 | 88.26 | 46.55 | 39 | – | – |
| Twamley et al., 2009 | D-KEFS - Switching subtests contrast scaled score*^1^ (accuracy) | 9.0 | 2.7 | 55 | 9.2 | 1.9 | 20 | + | – |
| Inhibition |  |  |  |  |  |  |  |  |  |
| Aupperle et al., 2012 | D-KEFS - Color-Word Interference Inhibition scaled score (accuracy) | 9.97 | 2.64 | 37 | 11.24 | 2.65 | 33 | + | – |
| Daugherty et al., 2018 | FDT-3 (accuracy) | 37.42 | 8.74 | 45 | 31.26 | 4.99 | 39 | – | – |
| Twamley et al., 2009 | D-KEFS - Color-Word Interference Test Inhibition vs. Color Naming scaled score (accuracy) | 11.1 | 2.3 | 55 | 11.3 | 1.8 | 20 | + | – |
| Working memory |  |  |  |  |  |  |  |  |  |
| Daugherty et al., 2018 | WAIS-III - Letters & Numbers (accuracy) | 8.67 | 2.72 | 45 | 10.49 | 3.47 | 39 | – | – |
| Semantic verbal fluency |  |  |  |  |  |  |  |  |  |
| Stein et al., 2002 | COWAT - Category Test (number of errors)*^2^ | 38.3 | 15.6 | 17 | 30.4 | 11.7 | 22 | + | – |

*Note.* IPV = Intimate partner violence; PTSD = Posttraumatic stress disorder; BI = Brain injury; *M* = Mean; *SD* = Standard deviation; *n* = Sample size; D-KEFS = Delis-Kaplan Executive Function System; MCST = Modified Card Sorting Test; TMT-4 = Trail Making Test - Part 4; D-KEFS - Switching subtests contrasting scores refer to the scaled scores from the TMT Number-Letter Switching vs. Combined Number Sequencing and Letter Sequencing Contrast Score; FDT-3 = Five Digit Test - Part 3; WAIS-III = Wechsler Adult Intelligence Scale - 3^rd^ Edition; COWAT = Controlled Oral Word Association Test.

+ With PTSD diagnosis or moderate/severe BI history; – Without PTSD diagnosis or moderate/severe BI history.

*^1^ Higher scores indicate better performance, in contrast with the other measures in the same domain; *^2^ Higher scores indicate worse performance in contrast with the other measures in the same domain; In these cases, the meta-analysis included the scores following their inversion.
